# Social memory maintenance relies on social interaction-induced proteolytic products of neuroligin 1

**DOI:** 10.1038/s41392-025-02467-6

**Published:** 2025-11-24

**Authors:** An Liu, Xingcan Li, Mei Zhuang, Qiaoyun Ren, Jinglei Zhang, Dandan Lv, Miao Wu, Xingjie Bian, Chengyan Zhu, Xiuqi Yang, Moyi Li, Yanan Wang, Zhengping Jia, Wei Xie

**Affiliations:** 1 The Key Laboratory of Developmental Genes and Human Disease, The School of Life Science and Technology, Southeast University, Nanjing, China; 2 Institute for Brain and Intelligence, Southeast University, Nanjing, China; 3 Neurosciences & Mental Health, The Hospital for Sick Children, Toronto, ON Canada; 4 Department of Physiology, Faculty of Medicine, University of Toronto, Toronto, ON Canada; 5 Jiangsu Co-innovation Center of Neuroregeneration, Southeast University, Nanjing, China

**Keywords:** Molecular neuroscience, Cellular neuroscience

## Abstract

Proper social behaviors are essential for survival and success, and deficits in these behaviors are associated with many brain disorders. However, the mechanisms underlying the formation and maintenance of social memory remain poorly understood. In this study, we demonstrate that social interaction with unfamiliar mouse induces α- and γ-secretase-dependent proteolysis of Neuroligin 1 (NLG1) in the ventral hippocampus (vHPC). The intracellular hydrolysate fragment, NLG1-CTD, regulates synaptic plasticity, spine strengthening, and the maintenance of social memory through its PDZ binding domain (PBD) and the cofilin signaling pathway. Both γ-secretase inhibition and deletion of the secretase recognition site on NLG1 prevent cofilin phosphorylation and impair the maintenance of social memory by inhibiting the production of NLG1-CTD. Injection of the Tat-PBD peptide into the vHPC inhibits cofilin activity and rescues deficits in social memory maintenance in mouse models. Additionally, our findings indicate that deficits in maintaining memory for sequentially presented social objects within a short temporal interval may be associated with insufficient levels of NLG1-CTD. Supplementation of Tat-PBD into the vHPC promotes maturation of dendritic spines and restores the maintenance of memory for the second social object. We also discovered that NLG1-CTD/PBD may play a role in maintaining novel object recognition memory. In summary, this work uncovers a novel mechanism that links extracellular and intracellular signal transduction processes to synaptic remodeling during learning and memory maintenance, providing a systematic perspective that connects memory formation, maintenance, and synaptic structural and functional plasticity.

## Introduction

The mystery of memory has intrigued humans for millennia. In terms of temporal scale, memory, especially declarative memory, can be divided into distinct forms, such as working memory, short-term memory, and long-term memory.^1–3^ We now know that the formation of short-term memory (seconds to minutes) depends on the phosphorylation of key proteins and synaptic plasticity within the limbic system, particularly the hippocampus.^4–6^ On the other hand, the storage of long-term memory (several hours or longer) is believed to be linked to gene transcription in critical cells, like engram cells, dispersed across various brain regions in the limbic system and neocortex.^4–7^ These studies provide a broad understanding of the physiological processes behind memory formation, both shortly before and several hours after learning. However, the mechanisms that maintain short-term memory, which lasts from tens of minutes to several hours, remain a puzzle.

Social memory refers to the ability of animals to remember individuals of the same species after engaging in social interactions. Deficits in social memory are associated with various mental disorders, including Alzheimer’s disease (AD), autism spectrum disorder (ASD), and schizophrenia.^8,9^ Key brain regions involved in short-term social memory formation include the dorsal hippocampal CA2 (dCA2) and the ventral hippocampus (vHPC).^9–12^ Inhibition of dCA2 or vHPC neuronal activity, along with the deletion of essential molecules like oxytocin, Shank3, as well as the Neuroligin 3 (NLG3) in astrocytes, has been shown to impair social memory formation.^10–14^ Moreover, prior research has identified social memory engram cells in the vCA1 region, and inhibiting these cells can prevent the retrieval of long-term social memory.^15^ These findings suggest that there are still sustained, self-organized physiological activities in the vHPC region after social behavior occurs. However, the precise cellular processes underlying this modulation remain unclear.

Our previous studies have shown that introducing a novel social stimulation activates α-secretases in the vHPC,^16^ which have been reported to cleave various type-I transmembrane proteins.^17,18^ Following the extracellular shedding by α-secretase, the proteolytic enzyme γ-secretase is recruited to perform sequential cleavage of the intracellular segments, generating a soluble short peptide fragment and an intracellular fragment.^19,20^ These findings suggest that the input of social information, via the activation of α-secretase-dependent protein cleavage, may induce complex and sustained effects on the vHPC, potentially contributing to the formation and maintenance of social memory. Identifying the key molecules or mechanisms from a vast array of secretase substrates is a challenging yet fascinating endeavor.

Neuroligin1 (NLG1) is a well-known glutamatergic excitatory postsynaptic adhesion molecule and is associated with ASD.^21–23^ It forms trans-synaptic connections with its presynaptic binding partner, Neurexins (NRXNs), to maintain synaptic stability and regulate synaptic function.^21,24^ Studies have shown that NLG1 knockout (KO) mice exhibit impairments in long-term potentiation (LTP), repetitive stereotyped behaviors, and deficits in social memory.^25,26^ Additionally, it has been reported that NLG1 undergoes activity-dependent proteolysis through sequential cleavage mediated by matrix metalloprotease 9 (MMP9) or α-secretase and γ-secretase, resulting in the production of soluble extracellular and intracellular fragments.^27,28^ Our previous research has demonstrated that the soluble intracellular fragment of NLG1 (NLG1-CTD) can regulate the LIMK/cofilin signaling pathway and promote dendritic spine formation and LTP maintenance through its carboxyl-terminal PDZ binding domain (PBD).^29^ However, the in vivo functions of NLG1 cleavage and NLG1-CTD, especially in cognitive behavior, remain unclear.

In this study, we demonstrate that social stimulation enhances synaptic transmission in the vHPC and promotes the proteolysis of NLG1. The resulting C-terminal fragment of NLG1 (NLG1-CTD) contributes critically to the maintenance of social memory via its PBD motif, which inhibits cofilin and thereby strengthens dendritic spines. Furthermore, we reveal that this mechanism underlies the impaired maintenance of memory for sequentially presented social stimuli with short intervals, as well as for object memory.

## Results

### The intracellular proteolytic by γ-secretase is involved in social memory maintenance

**Fig. 1:**
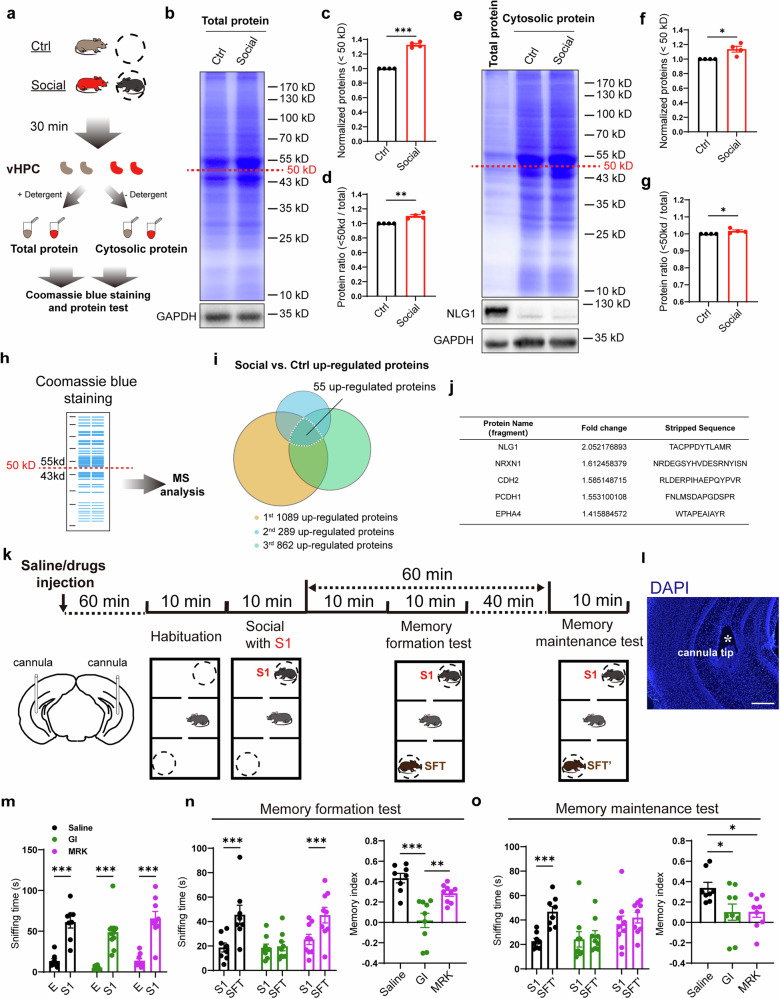
Social interaction promotes proteolysis in vHPC. **a** Illustration of collecting Ctrl and social groups of mouse vHPC total and cytosolic protein tissues for Coomassie blue staining and protein test; **b** Sample image of Coomassie blue staining of total vHPC proteins; **c**, **d** Summary data showing significantly increased <50 kDa proteins (**c**) and increased ratio of <50 kDa over total protein (**d**) in total vHPC tissues of social group (n = 4 mice); **e** Sample image of Coomassie blue staining of cytosolic vHPC proteins; **f**, **g** Summary data showing significantly increased <50 kDa proteins (**f**) and increased ratio of <50 kDa over total protein (**g**) in cytosolic vHPC tissues of social group (n = 4 mice); **h** Illustration of collecting <50 kDa proteins from gels after Coomassie blue staining for MS assay; **i** Summary graph of the 3 times of MS analyses showing 55 proteins (fragments) were increased in the social group; **j** List of 5 increased proteins (fragments) in (**i**); **k** Illustration of drugs in situ injection and mouse social memory formation and maintenance detection through modified 3-chamber experiments; **l** Representative image showing the site of cannula implant, scale bar: 200 μm; **m** Sniffing time detection showing the saline (n = 8 mice), GI (n = 9 mice) and MRK (n = 9 mice) injected mice preferred S1 over empty cage (E); **n** Sniffing time detection (left) and memory formation index (right) showing saline (n = 8 mice) and MRK (n = 9 mice), but not GI (n = 9 mice) injected mice preferred SFT over S1; **o** Sniffing time detection (left) and memory maintenance index (right) showing saline (n = 8 mice), but not GI (n = 9 mice) and MRK (n = 9 mice) injected mice preferred SFT’ over S1. Data represent mean ± SEM; two-tailed *t*-test for **c**, **d**, **g** and **h**; two-way ANOVA with Fisher’s LSD post hoc multiple comparisons for **m** and left panels of **n** and **o**; one-way ANOVA with Fisher’s LSD post hoc comparisons for right panels of **n** and **o**. *p < 0.05, **p < 0.01, ***p < 0.001. Also see Fig. S1

Social stimulation has been shown to activate various brain regions and cellular processes.^30,31^ Our previous research in mice demonstrated that the social interaction with novel mouse (stranger) increases the expression of brain α-secretase (mature ADAM10) in the vHPC region (Fig. S1a, b).^16^ Considering that there are hundreds of substrates for α-secretase,^17,32^ the activation of α-secretase induced by social stimulation may lead to alterations in the overall protein composition. To investigate this, we isolated proteins from the vHPC tissues of the mice subjected to social stimulation, and performed SDS-PAGE electrophoresis followed by Coomassie Blue staining experiment (Fig. 1a, b). The results indicated that after social stimulation, the total amount of vHPC proteins with the molecular weight less than 50 kDa significantly increased, as did the proportion of these proteins relative to the total protein content (Figs. 1c, d and S1c). Since the substrate proteins that undergo ectodomain shedding can be sequential cleaved by γ-secretase and produce soluble intracellular protein fragments, we also extracted cytoplasmic proteins and performed Coomassie Blue staining experiments to examine the intracellular protein levels (Fig. 1e). The results indicated that the proportion of cytoplasmic vHPC proteins with molecular weight of less than 50 kDa also significantly increased following social stimulation (Figs. 1f, g and S1d), suggesting an increase of intracellular proteolytic. To determine the molecular identity of increased protein components, we extracted total vHPC total proteins under 50 kDa from the PAGE gel for mass spectrometry analysis (Fig. 1h). The results revealed that 55 among over 2000 detected proteins (fragments) were upregulated (fold change > 1.2, Fig. 1i). Certain fragments originate from known substrates of α-and γ-secretases, such as NLG1, NRXN1, and CDH2 (Fig. 1j).^27,33,34^ Gene ontology (GO) enrichment analyses of these proteins showed that some of them were related to synaptic processes, such as synaptic vesicle cycle and postsynaptic membrane assembly (Fig. S1e), suggesting social interaction may induce synaptic reorganization and plasticity-like changes in vHPC. We performed bilateral injections of the α-secretase inhibitor GI254023X (GI) prior to social stimulation, followed by vHPC total proteins collection and Coomassie blue staining 30 min post-stimulation (Fig. S1f). The results revealed that the inhibition of ADAM10 impaired the social stimulation-induced increase in the proportion of proteins under 50 kDa (Fig. S1g–i).

To investigate whether the activity of α- and γ-secretases in the vHPC is involved in the formation and maintenance of social memory, we bilaterally implanted cannulas in the vHPC region of mice. After one week of recovery, we injected the saline, GI, and the γ-secretase specific inhibitor MRK-560 (MRK), respectively.^35,36^ One hour later, we conducted three-chamber social memory tests (Fig. 1k, l). A stranger mouse (S1) was introduced into one side-chamber after a 10-min habituation period. All three groups of mice spent more time exploring S1 (Fig. 1m), indicating that they exhibited normal social preference. The mice were then allowed to freely explore the middle-chamber for 10 min. Subsequently, a second stranger mouse, designated for the memory formation test (stranger for test, SFT), was placed into another side-chamber. As shown in Fig. 1n, both the saline and MRK-injected mice spent more time exploring the novel mouse (SFT), while the α-secretase inhibitor GI-injected mice did not, suggesting that the inhibition of α-secretase blocks social memory formation. To assess the effects of secretases on social memory maintenance to S1, we introduced a third stranger mouse (SFT’) and performed memory maintenance test 40 min after the formation test (60 min after the social interaction with S1) (Fig. 1k). As shown in Fig. 1o, the saline-injected mice exhibited preference for the novel mouse (SFT’), while both the GI and MRK-injected mice showed similar sniffing times for S1 and SFT’, indicating impaired social memory maintenance in these two groups of mice.

To investigate whether protein synthesis is involved in the maintenance of social memory, we also administered the protein synthesis inhibitor anisomycin 30 min prior to the social interaction with S1 (Fig. S1j), and the results indicated that anisomycin inhibited new protein synthesis, but did not affect the maintenance of social memory (Fig. S1k–m).

In summary, these results indicate that social stimulation promotes the proteolysis of α- and γ-secretase substrates in the vHPC. The formation of social memory relies on the activity of α-secretase, and the γ-secretase is specifically involved in the maintenance of social memory.

### Social stimulation promotes synaptic transmission in vHPC

**Fig. 2:**
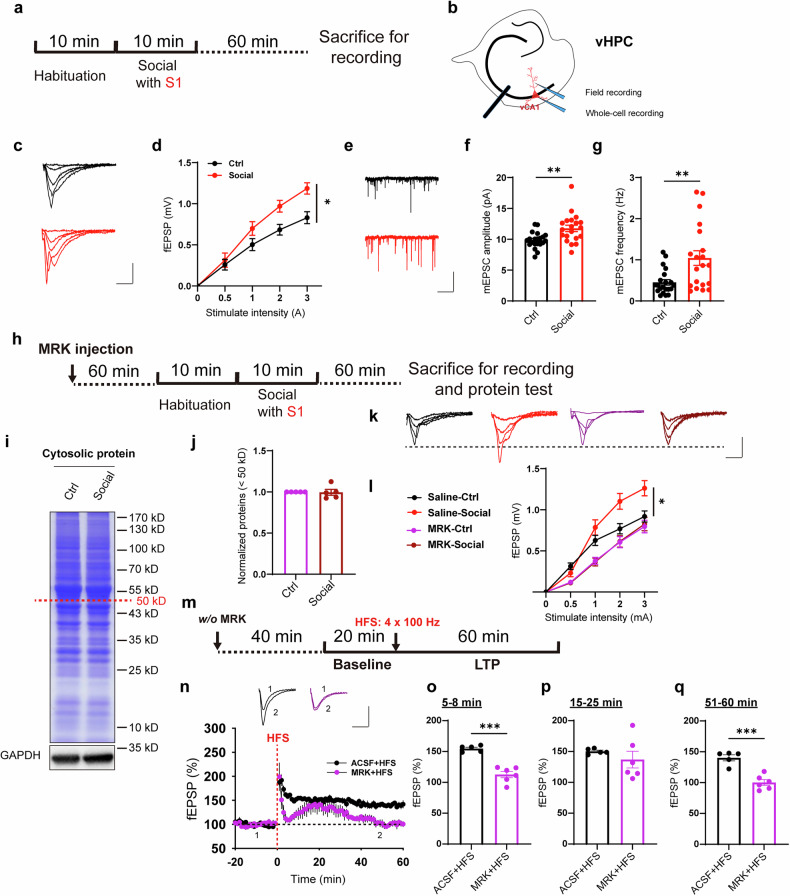
MRK blocks social induced synaptic transmission enhancement. **a**, **b** Illustration of field and whole-cell recording on vHPC slices; **c**, **d** Sample traces and summary input/output curve of fEPSPs showing increased synaptic transmission in social group (n = 14 slices from 5 mice) compared to the slices from Ctrl mice (n = 14 slices from 5 mice), scale bar: 0.5 mV/10 ms; **e**–**g** Sample traces and summary graphs showing increased mEPSC amplitude (**f**) and frequency (**g**) in the vCA1 neurons of social group (n = 20 cells from 5 mice) compared to that of Ctrl mice (n = 21 cells from 5 mice), scale bar: 10 pA/2 s; **h** Illustration of drug in situ injection and collecting mice vHPC tissues or slices for recording and protein test; **i** Sample image of Coomassie blue staining of cytosolic vHPC proteins after MRK pre-treatment; **j** Summary data showing comparable cytosolic vHPC <50 kDa proteins in MRK pre-treated Ctrl and social groups (n = 5 mice); **k,** **l** Sample traces and summary input/output curve of fEPSPs showing comparable synaptic transmission in Saline and MRK pre-treated Ctrl and social groups (n = 14 slices from 5 mice for each group; Saline-Ctrl vs. Saline-Social, F_(1,26)_ = 4.922, p < 0.05; MRK-Ctrl vs. MRK-Social, F_(1,26)_ = 0.0556, p = 0.815), scale bar: 0.5 mV/10 ms; **m** Illustration of MRK treatment and HFS induced LTP recording on vHPC slices; **n** Summary graphs showing impaired HFS induced LTP in MRK pre-treated vHPC slices (n = 6 slices from 3 mice) compared to that in Ctrl slices (n = 5 slices from 3 mice), scale bar: 0.5 mV/25 ms; **o**–**q** Summary graphs of (**n**) showing impaired early expression (**o**) and maintenance (**q**) of LTP, but normal middle-term LTP expression (**p**) in MRK pre-treated vHPC slices. Data represent mean ± SEM; two-tailed t-test for **f**, **g**, **j**, **o**, **p** and **q**; repeated two-way ANOVA with Fisher’s LSD post hoc multiple comparisons for **d** and **l**. *p < 0.05, **p < 0.01, ***p < 0.001. Also see Fig. S2

To investigate whether alterations in synaptic function in vHPC accompany the maintenance of short-term social memory, we examined synaptic transmission in ventral hippocampal slices from mice 60 mins after social stimulation. We positioned the stimulation electrode in the stratum radiatum (SR) layer of vCA1 and recorded the excitatory synaptic transmission induced by electrical stimulation in this area and vCA1 pyramidal neurons (Fig. 2a, b). The results demonstrated a significant increase in the field excitatory postsynaptic potential (fEPSP) following social stimulation (Figs. 2c, d and S2a). Subsequently, we conducted measurements of miniature postsynaptic currents. The results revealed a significant increase in both the amplitude and frequency of miniature excitatory postsynaptic currents (mEPSCs) (Fig. 2e–g). In contrast, the amplitude and frequency of miniature inhibitory postsynaptic currents (mIPSCs) remained intact (Fig. S2b–d). These findings indicate that mice exposed to social stimuli exhibit long-term potentiation (LTP)-like enhancements in excitatory synaptic transmission within the vCA1 pyramidal neurons. The activation of calcium–calmodulin (CaM)-dependent protein kinase II (CaMKII) and the phosphorylation of the AMPA receptor subunit GluA1 at the serine 831 site (S831) are considered hallmarks of LTP formation and expression.^37,38^ We subsequently measured the phosphorylation levels of these proteins in the vHPC tissues at various time points following social stimulation using Western blotting (WB) (Fig. S2e). The results demonstrated that phosphorylation of both CaMKII and GluA1-S831 was significantly increased after social behavior (Fig. S2f–h). To investigate the impact of CaMKⅡ on ADAM10 activation and the formation and maintenance of social memory, we bilaterally injected the CaMKⅡ inhibitor KN-62 into the vHPC regions (Fig. S2i). The results demonstrated that social stimulation no longer induced an increase in mature ADAM10 (Fig. S2n, o), and the formation of social memory was impaired (Fig. S2j, k). These findings suggest that both the maturation of ADAM10 and the formation of social memory depend on CaMKⅡ activity. We further examined the role of CaMKⅡ in the maintenance of social memory by administering KN-62 into the vHPC of mice immediately after social stimulation. The results revealed that the maintenance of social memory was also impaired (Fig. S2l, m), indicating that CaMKⅡ is persistently involved in the maintenance of memory.

To determine whether enhanced excitatory synaptic transmission is associated with the activity of γ-secretase, we injected the γ-secretase inhibitor MRK-560 into the ventral hippocampus of mice before social stimulation. One hour after the social stimuli, we conducted Coomassie Blue staining and electrophysiological recordings (Fig. 2h). The results indicated that following MRK injection, social stimulation no longer resulted in an increase in cytosolic protein levels (Fig. 2i, j) or enhanced synaptic transmission (Figs. 2k, l and S2p) in the vCA1 region. However, the phosphorylation levels of both CaMKⅡ and GluA1-S831 were still increased one hour after social behavior (Fig. S2q–s), suggesting these processes were not γ-secretase activity dependent. To further investigate the effect of MRK on synaptic transmission and plasticity in the vHPC, we first performed field potential recordings in the SR region of vCA1. The results showed that 5 μM MRK significantly suppressed electrically evoked fEPSPs (Fig. S2t, u). Next, we pre-incubated ventral hippocampal slices with MRK for one hour and induced LTP in the vCA1 SR region using high-frequency electrical stimulation (HFS, 4 × 100 Hz) (Fig. 2n). As shown in Fig. 2o–q, abnormal early and late expressions of HFS-LTP were observed in the slices treated with MRK. Although an enhancement in synaptic transmission was observed around 20 min after stimulation (Fig. 2p), it could not be sustained (Fig. 2q). These results suggest that the enhancement of synaptic transmission in the ventral hippocampus induced by social stimulation and high-frequency electrical stimulation is dependent on the activity of γ-secretase.

### Inhibiting NLG1 in ventral hippocampal neurons impairs social memory maintenance

Next, we asked the key substrate of γ-secretase that regulate these processes. Previous studies have demonstrated that NLG1 serves as a substrate for both α- and γ-secretase.^27,29^ Its intracellular proteolytic fragment can regulate dendritic spine formation and LTP amplitude through its carboxyl-terminal PBD sequence.^29^ Additionally, we observed an increase in NLG1-CTD levels in the MS experiments of vHPC protein tissue following social interaction (Fig. 1i). Therefore, we hypothesize that the accumulation of NLG1-CTD, may play a crucial role in maintaining social memory and enhancing synaptic transmission.

**Fig. 3:**
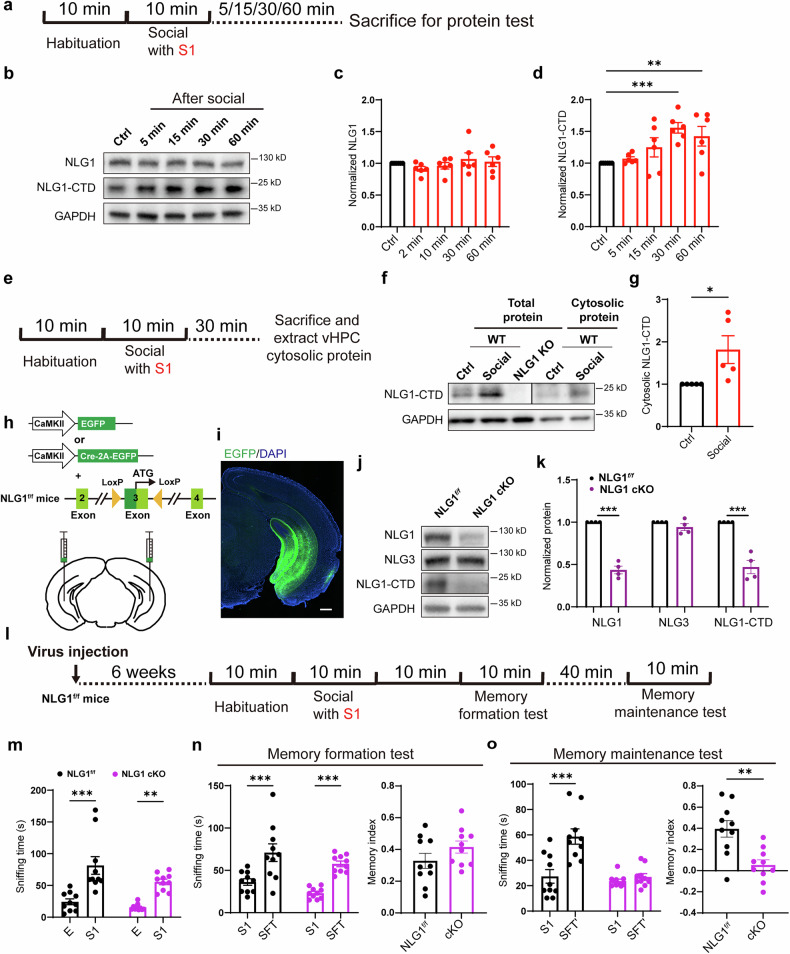
vHPC NLG1 undergoes proteolysis and is necessary for social memory maintenance. **a** Illustration of collecting vHPC proteins at different time points after social interaction; **b**–**d** Sample images and summary graphs (n = 6) showing social promotes NLG1-CTD generation (**d**), but has less effect on full length NLG1 protein level (**c**); **e** Illustration of collecting vHPC cytosolic proteins 30 mins after social interaction; **f**, **g** Sample images and summary graph showing social promotes cytosolic NLG1-CTD generation; **h**, **i** Illustration and sample image showing injecting and expressing AAV viruses in vHPC of NLG1^*f/f*^ mice, scale bar: 200 μm; **j**, **k** Sample images and summary graphs showing decreased full-length NLG1 and NLG1-CTD, but not NLG3 in NLG1 cKO mice (n = 4); **l** Illustration of virus injection and social memory formation and maintenance detection in NLG1^*f/f*^ male mice; **m** Sniffing time detection showing both the male NLG1^*f/f*^ (n = 10) and NLG1 cKO (n = 10) mice preferred S1 over empty cage (E); **n** Sniffing time detection (left) and memory formation index (right) showing both the male NLG1^*f/f*^ (n = 10) and NLG1 cKO (n = 10) mice preferred SFT over S1; **o** Sniffing time detection (left) and memory maintenance index (right) showing the male NLG1^*f/f*^ (n = 10), but not NLG1 cKO (n = 10) mice preferred SFT’ over S1. Data represent mean ± SEM; two-tailed t-test for **g**, **k** and right panels of **n** and **o**; one-way ANOVA with Fisher’s LSD post hoc comparisons for right panels of **c** and **d**; two-way ANOVA with Fisher’s LSD post hoc multiple comparisons for **m** and left panels of **n** and **o**. *p < 0.05, **p < 0.01, ***p < 0.001. Also see Figs. S3 and S4

To verify the MS results, we measured the levels of NLG1-CTD protein fragments in the ventral hippocampal tissue at various time points following social stimulation (Fig. 3a). The results indicated that 30 and 60 min after social stimuli, the levels of NLG1-CTD, but not full-length NLG1 were significantly higher than those in the control group (Fig. 3b–d). The antibody we used specifically against the intracellular domain of NLG1 and has been validated in NLG1 KO samples for detecting NLG1-CTD.^27–29^ Additionally, we extracted cytoplasmic vHPC proteins 30 min after the social interaction and performed WB analysis (Fig. 3e). The results demonstrated that the protein levels of cytoplasmic NLG1-CTD were significantly elevated in the tissue that had undergone social stimulation compared to the control group (Fig. 3f, g).

To determine whether NLG1 deficiency in vCA1 neurons impairs the maintenance of social memory, we bilaterally injected excitatory neuron-specific CaMKⅡ-driven Cre (rAAV-CaMKⅡ-CRE-2A-EGFP) virus into the vHPC regions of floxed *Nlg1* mice (NLG1^*f/f*^)^23^ (Fig. 3h, i). To ensure the endogenous NLG1 protein was fully degraded, protein and behavioral tests were conducted 6 weeks after the viral injection. The protein test results showed that both full-length NLG1 and NLG1-CTD were significantly reduced, while full-length NLG3 did not (Fig. 3j, k). Next, we performed 3-chamber behavioral tests to measure the social memory formation and maintenance ability of both male and female NLG1 conditional KO (cKO) mice (Figs. 3l and S3a). The results revealed that KO NLG1 in vHPC of excitatory neurons did not disrupt social preference or memory formation in either gender (Figs. 3m, n and S3b, c). However, it did impair the maintenance of social memory in both male and female mice (Figs. 3o and S3d), suggesting that NLG1 is specifically essential for the maintenance of social memory. We next administered AAV viruses with CaMKII promoter-driven Cre (rAAV-CaMKII-CRE) and Cre-dependent NLG1-shRNA (rAAV-CMV-bGlobin-Flex-EGFP-MIR30shRNA(NLG1)) to achieve knockdown (KD) NLG1 in male WT vHPC excitatory neurons (Fig. S3e). The results indicated that the expression of the NLG1 KD virus effectively reduced both the full-length NLG1 and NLG1-CTD levels (Fig. S3f–h), and blocked social memory maintenance, but not memory formation (Fig. S3i–l). Additionally, electrophysiological recording experiments demonstrated that inhibiting NLG1 impaired the social induced synaptic transmission enhancement in vCA1 region (Fig. S3m–o).

The mass spectrometry results indicated that the intracellular domain of NRXN1 was present in the elevated intracellular fraction, which serves as a crucial presynaptic binding partner of NLG1.^21,24^ Consequently, we investigated its role in social memory using floxed *Nrxn1* mice (NRXN1^*f/f*^). We bilaterally injected excitatory neuron-specific CaMKⅡ-driven Cre (rAAV-CaMKⅡ-CRE-2A-EGFP) virus into the vHPC regions to generate Nrxn1 cKO mice (Fig. S4a–d). Interestingly, we also detected a reduction in the protein levels of both full-length NLG1 and NLG1-CTD in NRXN1 cKO tissues (Fig. S4c, d). Furthermore, we found that NRXN1 deletion in mice vHPC neurons did not impair the formation of short-term social memory (Fig. S4e, f), but modestly impaired its maintenance (Fig. S4g). These results indicate that NRXN1 in the vHPC participates in regulating social memory maintenance, which may be associated with the reduction in NLG1 and NLG1-CTD. Subsequently, protein samples from the vHPC of NLG1 cKO mice, both with and without social stimulation, were subjected to CB staining and MS analysis (Fig. S4h). The CB staining results indicated that inhibiting NLG1 in vHPC excitatory neurons did not prevent the social experience-induced increase in protein fragments below 50 kDa (Figs. S4i, j). From triplicate MS analyses, we identified 76 upregulated proteins (Fig. S4k). Among these, fragments of NRXN1 and CDH2, identified via intracellular peptide sequences, remained detectable. However, for the stochastic nature of MS detection, fragments of PCDH1 and EPHA4 were not detected this time. Nonetheless, an increase in the intracellular fragment of Dystroglycan 1 (DAG1),^39^ another secretase substrate, was observed (Fig. S4l). These results suggest that the loss of NLG1 in vHPC excitatory neurons does not affect the social experience-induced proteolysis of other secretase substrates, while concurrently providing further support for the correlation between NLG1 proteolysis and the maintenance of social memory.

In summary, our results indicate that social stimuli promote the proteolysis of NLG1 in the vHPC region, while inhibiting NLG1 prevents the maintenance of short-term social memory without affecting its formation.

### The cleavage of NLG1 promotes social memory maintenance

**Fig. 4:**
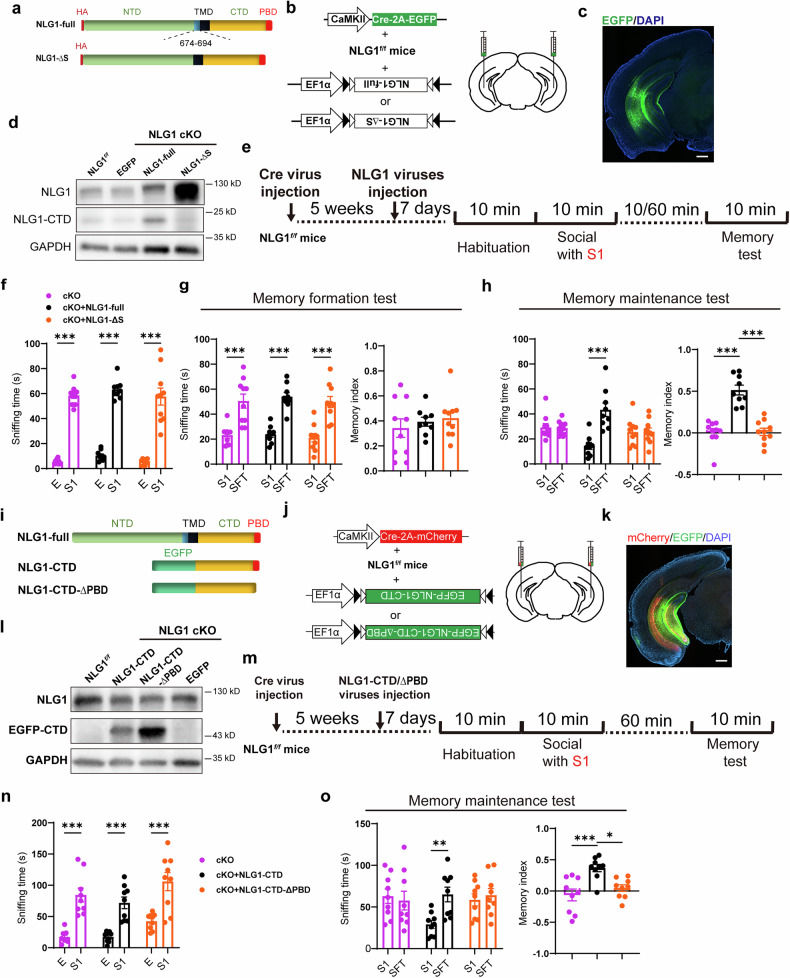
Social memory maintenance depends on NLG1 proteolysis and NLG1-CTD generation. **a** Illustration diagrams of NLG1 full-length and NLG-ΔS proteins; **b**, **c** Illustration and sample image showing injecting and expressing NLG1 full-length and NLG-ΔS in vHPC of NLG1cKO mice, scale bar: 200 μm; **d** Sample images demonstrating the expression of NLG1 full-length, NLG-ΔS and NLG1-CTD proteins; **e** Illustration of viruses injection and social memory formation and maintenance detection; **f** Sniffing time detection showing all the NLG1 cKO (n = 10), NLG1-full (n = 9) and NLG-ΔS (n = 10) expressed NLG1 cKO mice preferred S1 over empty cage (E); **g** Sniffing time detection (left) and memory formation index (right) showing all the NLG1 cKO (n = 10), NLG1-full (n = 9) and NLG-ΔS (n = 10) expressed NLG1 cKO mice preferred SFT over S1; **h** Sniffing time detection (left) and memory maintenance index (right) showing the NLG1-full (n = 9), but not NLG-ΔS (n = 10) expressed NLG1 cKO mice preferred SFT’ over S1; **i** Illustration diagrams of NLG1-full, NLG1-CTD and NLG-CTD-ΔPBD proteins; **j**, **k** Illustration and sample image showing injecting and expressing NLG1-CTD and NLG-CTD-ΔPBD in vHPC of NLG1cKO mice, scale bar: 200 μm; **l** Sample images demonstrating the expression of NLG1-CTD and NLG-CTD-ΔPBD proteins in different group of mice; **m** Illustration of viruses injection and social memory maintenance detection; **n** Sniffing time detection showing all the NLG1 cKO (n = 9), NLG1-CTD (n = 9) and NLG-CTD-ΔPBD (n = 9) expressed NLG1 cKO mice preferred S1 over empty cage (E); **o** Sniffing time detection (left) and memory maintenance index (right) showing the NLG1-CTD (n = 9), but not NLG-CTD-ΔPBD (n = 9) expressed NLG1 cKO mice preferred SFT’ over S1. Data represent mean ± SEM; two-way ANOVA with Fisher’s LSD post hoc multiple comparisons for **f**, **n** and left panels of **g**, **h** and **o**; one-way ANOVA with Fisher’s LSD post hoc comparisons for right panels of **g**, **h** and **o**. *p < 0.05, **p < 0.01, ***p < 0.001. Also see Fig. S5

To investigate whether NLG1 proteolysis is essential for maintaining social memory, we constructed a mutant NLG1 (NLG1-ΔS) with the sequence from positions 674 to 694 deleted (Fig. 4a). This deletion prevents recognition by ADAM10 for sequential proteolysis,^27^ while preserving its ability to be transported to the cell membrane (Fig. S5a, b). We then injected either the mutant NLG1 (AAV-HA-NLG1-ΔS) or WT NLG1 (AAV-HA-NLG1-full) virus into the ventral hippocampus of NLG1 cKO mice (Fig. 4b–d). Results from the 3-chamber social experiments indicated that overexpression of NLG1-full and NLG1-ΔS did not impact social preference or the formation of short-term social memory (Fig. 4f, g). However, NLG1-full, not NLG1-ΔS, was able to restore the maintenance of social memory following NLG1 inhibition (Fig. 4h). These findings suggest that NLG1 proteolysis is crucial for the maintenance of social memory.

Subsequently, we aimed to determine the role of intracellular proteolysis product NLG1-CTD in maintaining social memory. Our previous research indicated that NLG1-CTD facilitates synaptic transmission and LTP through its carboxyl-terminal PBD sequence.^29^ To investigate this further, we constructed a virus expressing the intracellular fragment of NLG1 (AAV-DIO-NLG1-CTD) and a mutant version of the intracellular fragment (AAV-DIO-NLG1-CTD-ΔPBD), which lacks the PBD sequence (Fig. 4i). We injected these viruses separately into the vHPC of NLG1 cKO mice (Fig. 4j–l) and performed social memory test 1 week later (Fig. 4m). The behavioral results demonstrated that the expression of NLG1-CTD, but not NLG1-CTD-ΔPBD, rescued deficits in social memory maintenance in vHPC NLG1 cKO mice (Fig. 4n, o). We also assessed the memory performance of NLG1 cKO mice at 4 h and 24 h following social stimulation (Fig. S5c). The results revealed cognitive deficits at both time points (Fig. S5d, e), indicating that NLG1 deletion impairs memory over extended durations. Subsequently, we reintroduced NLG1-CTD expression in the same cohort of mice and retested their social memory retention one week later (Fig. S5f). The results demonstrated that the memory deficits observed at both the 4-h and 24-h post-stimulation intervals were rescued (Fig. S5g, h).

### PBD peptide inhibits cofilin activity and participates in social memory maintenance

**Fig. 5:**
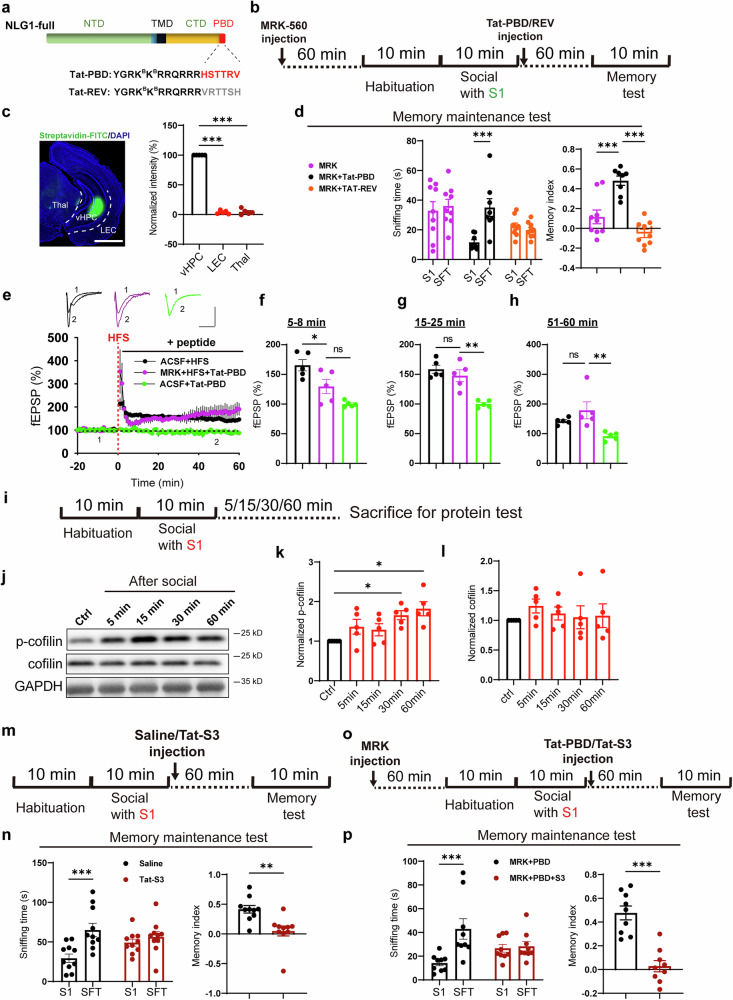
Social memory maintenance depends on PBD sequence and cofilin inactivation. **a** Illustration diagrams of biotinylated Tat-PBD and Tat-REV peptides; **b** Illustration of MRK and viruses injection and social memory maintenance detection; **c** Sample image and summary graph showing the Tat-PBD peptides restricted to vHPC region, scale bar: 500 μm; **d** Sniffing time detection (left) and memory maintenance index (right) showing the Tat-PBD (n = 8), but not Tat-REV (n = 10) and MRK alone (n = 9) injected mice preferred SFT’ over S1; **e** Summary curves showing comparable HFS induced LTP in Tat-PBD perfused-MRK pre-treated vHPC slices (n = 5 slices from 3 mice) compared to that in Ctrl slices (n = 5 slices from 3 mice), as a control, Tat-PBD alone could not cause any alteration of fEPSP, scale bar: 0.5 mV/25 ms; **f**–**h** Summary graphs of (**e**) showing impaired early expression (**f**), but normal middle-term (**g**) and maintenance of LTP (**h**) in MRK pre-treated vHPC slices; **i** Illustration of collecting vHPC proteins at different time points after social; **j**–**l** Sample images and summary graphs (n = 5) showing increased p-cofilin (**k**), but not total cofilin level (**l**) in vHPC tissues after social interaction; **m** Illustration of Tat-S3 peptide injection and social memory maintenance detection; **n** Sniffing time detection (left) and memory maintenance index (right) showing saline (n = 10), but not Tat-S3 (n = 10) injected mice preferred SFT over S1. **o** Illustration of MRK and peptides injection and social memory maintenance detection; **p** Sniffing time detection (left) and memory maintenance index (right) showing Tat-PBD (n = 9), but not Tat-S3 + Tat-PBD (n = 9) injected MRK pre-treated mice preferred SFT over S1. Data represent mean ± SEM; two-tailed t-test for right panels of **n** and **p**; one-way ANOVA with Fisher’s LSD post hoc comparisons for **c**, **f**, **g**, **h**, **k**, **l** and right panel of **d**; two-way ANOVA with Fisher’s LSD post hoc multiple comparisons for left panels of **d**, **n** and **p**. *p < 0.05, **p < 0.01, ***p < 0.001. Also see Fig. S6–7

The above experiments indicated that the PBD motif at the carboxyl- terminal of NLG1 is crucial for maintaining social memory. To further investigate its function, we designed two biotinylated cell membrane permeable peptides, incorporating the Tat sequence from HIV preceding the PBD amino acid sequence (Tat-PBD) and reversed PBD sequence (Tat-REV) (Fig. 5a). To mimic the generation of NLG1-CTD after social behavior, we bilaterally injected the peptides (50 nM, 1 μL per side) into the ventral hippocampus of MRK pre-treated mice right after their social interaction with S1 (Fig. 5b). The post-check with streptavidin-conjugated FITC revealed that one hour after injection, the Tat-PBD peptide was predominantly distributed in the CA1 SR region of the vHPC, with less signal was observed in adjacent regions such as the lateral entorhinal cortex (LEC) or thalamus (Thal) (Fig. 5c). When the peptide concentration was increased to 10 times the working concentration (500 nM), its presence was clearly detected signal within the dendrites. However, still very less peptide signal was observed in the LEC or Thal regions (Fig. S6a–c). The memory test revealed that injecting the Tat-PBD peptide immediately, but not 30 min after social interaction, rescued the inhibitory effect of MRK on the maintenance of social memory (Figs. 5c, d and S6d, e). Furthermore, administration of the Tat-PBD peptide immediately after social contact similarly rescued social memory maintenance deficit in vHPC-specific NRXN1 cKO mice (Fig. S6f).

We have shown that in vHPC slices, HFS-induced LTP, specifically the early (5-8 mins) and maintenance (51-60 mins) stages were blocked by MRK (Fig. 2n–q). To examine whether the PBD peptide was able to rescue the inhibitory effect of MRK on HFS-LTP, we added 10 μM Tat-PBD peptide into the perfused ACSF right after HFS delivery. The results showed that the Tat-PBD peptide rescued LTP maintenance (Fig. 5e, h), but not the early phase of LTP expression (Fig. 5e, f). These findings suggest that the PBD peptide mimics NLG1-CTD and is sufficient for the maintenance of LTP and social memory.

Next, we asked the downstream molecular mechanisms. Our previous research has demonstrated that NLG1-CTD strengthens synaptic transmission by promoting the phosphorylation of the F-actin regulatory protein cofilin, thereby inhibiting its severing activity.^29,40^ We validated the negative correlation between NLG1-CTD and cofilin phosphorylation in NLG1 cKO mice. First, we observed decreased levels of phosphorylated cofilin (p-cofilin) in the vHPC tissue of NLG1 cKO mice (Fig. S6g, h) and MRK incubated vHPC slices (5 μM, 1 h, Fig. S6i, j), whereas tissues expressing NLG1-CTD via AAV exhibited increased cofilin phosphorylation level (Fig. S6k, l). Next, by incubating the vHPC slices of NLG1 KO mice with the Tat-PBD peptide, we found that the Tat-PBD peptide alone was sufficient to enhance cofilin phosphorylation (Fig. S6m, n). We then investigated whether the maintenance of social memory also relies on changes in cofilin activity. To this end, we measured the phosphorylation levels of cofilin in the vHPC at various time points following social interaction (Fig. 5i). The results indicated that p-cofilin, but not total cofilin levels, increased after social behavior (Fig. 5j–l), suggesting that the maintenance of social memory is accompanied by the decrease in cofilin activity (increase in phosphorylation). Furthermore, we conducted examinations in vHPC NLG1 cKO mice that had undergone social interaction. The results showed that after knocking out NLG1 in excitatory neurons in the vHPC, social stimulation failed to promote cofilin phosphorylation but did not affect the phosphorylation of CaMKⅡ and GluA1 S831 (Fig. S6o, p). This suggests that NLG1 specifically regulates cofilin activity, rather than CaMKⅡ activity or the LTP related-phosphorylation of AMPAR.

The reduced activity of cofilin in neurons is believed to be associated with increased maturity of dendritic spines. Based on this premise, we investigated the effects of social stimulation on dendritic spine structure in vCA1 neurons. The rAAV9-NCSP-YFP-2E5 virus, which is capable of sparse neuronal labeling, was injected into the vHPC region of WT mice (Fig. S6q, r). Three weeks after viral expression, the mice underwent social stimulation, in vitro imaging and analysis were conducted on the morphology of dendritic spines along the secondary dendrites of vCA1 pyramidal neurons, one hour after stimulation. The results indicated increased dendritic spine densities and a higher proportion of mature spines (mushroom and stubby types) in the mice that had undergone social interaction (Fig. S6s, t), which correspond with our observation of elevated cofilin phosphorylation and enhanced synaptic transmission.

To investigate the effect of preventing cofilin inhibition on social memory, we bilaterally injected the cofilin activation peptide Tat-S3 (prevents phosphorylation^41^) into the ventral hippocampus of WT mice immediately after the social interaction and got blocked social memory maintenance (Fig. 5m, n). This result suggests that the maintenance of social memory requires cofilin inactivation. Next, we asked whether the role of NLG1-CTD/PBD on social memory maintenance was dependent on cofilin inactivity. As shown in Fig. 5o, p, we found the rescue effect of Tat-PBD on MRK-induced memory deficit was obstructed by the simultaneous injection of the Tat-S3 peptide. These findings suggest that the function of the PBD peptide in maintaining social memory relies on the decrease in cofilin activity.

Given that dCA2 pyramidal neurons are critical for the formation of short-term social memory,^10^ and considering our previous finding that social-induced upregulation of ADAM10 in the vHPC is modulated by dCA2 neuronal activity,^15^ we hypothesized that social experience-induced NLG1 proteolysis and cofilin phosphorylation in the vHPC depend on dCA2 neuronal activity. We bilaterally injected a virus expressing a floxed, engineered M4 muscarinic receptor into the dCA2 region of Amigo2-Cre mice, which specifically target hippocampal CA2 pyramidal neurons. Clozapine-N-oxide (CNO) was administered intraperitoneally 30 min prior to social stimulation (Fig. S7a, b). The results demonstrated that inhibition of dCA2 pyramidal neurons impaired both the formation and maintenance of social memory (Fig. S7c–e). Furthermore, social stimulation no longer increased the levels of NLG1-CTD, ADAM10, or cofilin phosphorylation (Fig. S7f–h). Consistent with this, CB staining revealed intact protein fragments below 50 kDa (Fig. S7i, j). These findings indicate that social experience-induced proteolysis, changes in cofilin activity, and social memory formation and maintenance are all regulated by dCA2 neuronal activity.

### PBD peptide restores deficit in social memory maintenance in sequential social tasks

**Fig. 6:**
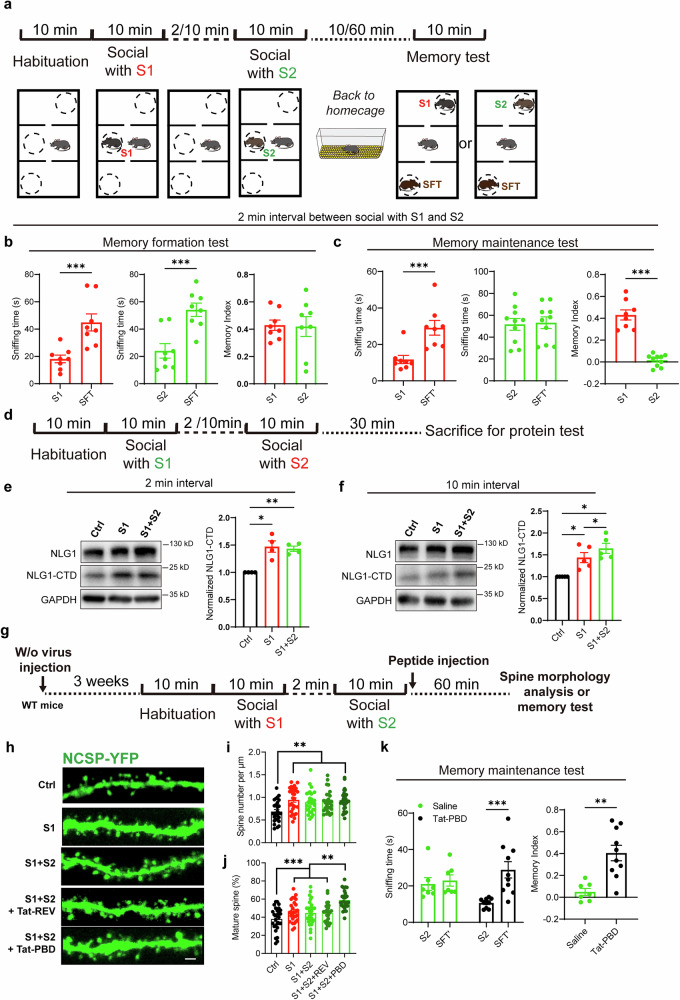
PBD peptide restores memory maintenance deficit in temporally close social tasks. **a** Illustration of continuous social tasks and social memory formation and maintenance detection through modified 3-chamber experiments; **b** Sniffing time detection and memory formation index (right) showing the subject mice (n = 8) preferred SFT over S1 (red) or S2 (green) in the 2 min interval continuous social task; **c** Sniffing time detection and memory maintenance index (right) showing the subject mice (n = 8) preferred SFT’ over S1 (red), but not S2 (green) in the 2 min interval continuous social task; **d** Illustration of collecting vHPC proteins from mice that have undergone continuous social tasks; **e** Sample images and summary graph showing a similar degree of elevation in the S1-only and 2 min-interval S1 + S2 groups of NLG1-CTD protein level, compared with that in the Ctrl group (n = 4); **f** Sample images and summary graph showing a higher degree of elevation of NLG1-CTD protein level in the 10 min interval S1 + S2 group than S1-only group, compared with that in the Ctrl group (n = 5); **g** Illustration of peptide injection and spine morphology and social memory detection after 2 min interval continuous social task; **h** Sample image of YFP expressed vCA1 dendrites in Ctrl, S1, S1 + S2, S1 + S2+Tat-REV and S1 + S2+Tat-PBD groups, scale bar: 2 μm; **i, j** Sample images and summary graphs showing compared to the Ctrl group (n = 28 dendrites from 12 neurons of 4 mice), elevated spine density and mature spine ratio in S1 (n = 28 dendrites from 12 neurons of 4 mice), S1 + S2 (n = 28 dendrites from 12 neurons of 4 mice) and S1 + S2+Tat-REV (n = 28 dendrites from 12 neurons of 4 mice) groups of vCA1 neurons, and S1 + S2+Tat-PBD (n = 28 dendrites from 12 neurons of 4 mice) group exhibited further increased the spine maturity, scale bar: 2 μm; **k** Sniffing time detection (left) and memory maintenance index (right) showing Tat-PBD (n = 9), but not saline (n = 8) injected mice preferred SFT’ over S2. Data represent mean ± SEM; paired two-tailed t-test for the first two panels of **b** and **c**; two-tailed t-test for the last panels of **b**, **c** and **k**; one-way ANOVA with Fisher’s LSD post hoc comparisons for **e**, **f**, **i** and **j**; two-way ANOVA with Fisher’s LSD post hoc multiple comparisons for left panel of **k**. *p < 0.05, **p < 0.01, ***p < 0.001. Also see Fig. S8

When meeting or learning items in a sequence, people tend to have a stronger impression of the first, while weak memory of the subsequent or middle items, which is the primacy effect of the serial position effects.^42–44^ To investigate whether similar phenomena occur in the context of serial social tasks involving mice, we allowed the subject mice to sequentially interact with two separate stranger mice (S1 and S2) at 2 min or 10-min intervals, and then assessed their social memory formation (10 min later) and maintenance (60 min later) (Fig. 6a). The results indicated that the subject mice were able to form and maintain social memory for both unfamiliar mice when the interval was 10 mins (Fig. S8a, b). However, when the interval between the two social interactions was only 2 min, the subject mice could form short-term social memory for both mice (Fig. 6b) but were unable to retain the memory for S2 (Fig. 6c).

To determine whether the deficiency in memory maintenance is related to the insufficient production of NLG1-CTD, we examined the protein levels of NLG1-CTD in the vHPC of the subject mice after the sequential social tasks (Fig. 6d). The results indicated that when the interval between exposure to S1 and S2 was 2 min, the NLG1-CTD level in the S1 + S2 group was similar to that in the S1-only group, and both were significantly higher than that in the non-social group (Fig. 6e). In contrast, the NLG1-CTD level in the S1 + S2 group was higher than that in S1-only group of mice, if the interval is 10 mins (Fig. 6f). We also conducted electrophysiological recordings in the vCA1 region of the mice following the 2 interval social tasks (Fig. S8c). The results demonstrated that the synaptic transmission efficiency was similar between the S1 + S2 and the S1-only group (Fig. S8d–h).

These results suggest that if the interval between exposure to two different social objects is too short, it may hinder the generation of NLG1-CTD, which may result in impairment in both the strengthening of synaptic transmission and memory maintenance for the second social object. To test this hypothesis, we administered the Tat-PBD peptide immediately after exposure to the S2 mouse in the 2-minute interval group and subsequently assessed dendritic spine morphology and the maintenance of social memory one hour later (Fig. 6g). The results showed that social interaction significantly increased the density and maturity of dendritic spines in vCA1 neurons compared to control mice. However, no further increase was observed in the S1 + S2 group relative to the S1-only group (Fig. 6h, i). In contrast, administration of the Tat-PBD peptide led to an additional increase in the proportion of mature dendritic spines (Fig. 6j). Behaviorally, Tat-PBD injection rescued the deficit in social memory maintenance for S2 (Fig. 6k). Together, these findings indicate that impaired memory maintenance for the second mouse in temporally close may be attributable to insufficient NLG1-CTD/PBD availability, leading to a plateau in spine structural plasticity.

### PBD peptide restores novel object memory maintenance in NLG1 defict mice

Next, we aimed to determine whether NLG1-CTD/PBD is involved in other forms of memory maintenance. The dorsal hippocampal CA1 (dCA1) region has been reported to participate in novel object recognition (NOR) but not in social memory formation.^15,45^ Additionally, we observed increased levels of NLG1-CTD and cofilin phosphorylation in the dorsal hippocampus after the mice explored a novel object (Fig. S9a–c), suggesting that NLG1-CTD/PBD may also contribute to dCA1-dependent NOR memory. To test this hypothesis, we conditionally knocked out NLG1 in the excitatory neurons of the dCA1 and performed NOR tests (Fig. S9d–g). The results showed that one hour after training, the mice failed to exhibit a preference for the novel object (Fig. S9h), indicating a deficit in NOR. Subsequently, we administered the Tat-PBD peptide via cannula-guided delivery to the same cohort of mice. This intervention restored their preference for the novel object (Fig. S9i, j).

These findings suggest that object memory may similarly involve the proteolysis of NLG1, and that the resulting NLG1-CTD/PBD fragment may conservatively play a role in the maintenance of object memory.

## Discussion

Compared to the extensively studied mechanisms underlying its induction, the processes maintain LTP and short-term memory remain poorly understood. In this study, we demonstrate that social interaction in mice triggers α- and γ-secretase-dependent cleavage of NLG1 in the vHPC, a process mediated by dCA2 pyramidal neurons and vHPC CaMKⅡ activity. The resulting soluble NLG1-CTD promotes dendritic spine strengthening by inhibiting cofilin activity via its PDZ-binding domain, thereby maintaining LTP and social memory (Fig. S8i). Further experiments reveal that this mechanism also supports memory retention in novel object recognition and multiple-social object discrimination tasks.

At first, we found that the protein components with a molecular weight of less than 50 kDa significantly increased in both the total and intracellular lysates of the vHPC following social stimulation. Mass spectrometry analysis revealed that the increased component included a number of fragments from α-secretase and γ-secretase substrates, indicating enhanced activity of these enzymes. Consistent with this, our previous study reported that social interaction can promote the maturation of α-secretase in an NMDA receptor activity-dependent manner.^16^ Many type I transmembrane proteins have been identified as substrates for these two secretases,^17–20^, and although some of them were not detected by our MS assay, such as APP and Notch, we can also speculate that social stimuli can promote the cleavage of these molecules and activate their downstream cellular processes or physiological pathways.^46,47^ In this study, we focused on the crucial role of NLG1 cleavage and its proteolytic product in the maintenance of social memory. First, we found that both the conditional KO and KD of NLG1 in the ventral hippocampal excitatory neurons impaired the maintenance of social memory without affecting the formation of short-term social memory. Secondly, we reintroduced the NLG1 cKO mice with an NLG1 mutant that could not undergo α-secretase-dependent proteolysis. The mutant NLG1 failed to rescue the deficit in social memory maintenance, whereas the re-expression of both NLG1 and NLG1-CTD did. Subsequently, we discovered that the deletion of the PBD sequence eliminated the rescue effect of NLG1-CTD, and the PBD peptide itself could reverse the social memory maintenance deficiency caused by γ-secretase inhibition. These results indicate that the PBD peptide is both sufficient and necessary for social memory maintenance. Finally, our study demonstrated that the phosphorylation level of cofilin in the ventral hippocampus significantly increased following social stimulation, and blocking cofilin phosphorylation inhibited social memory maintenance both in WT mice and the Tat-PBD rescued mice. These findings suggest that NLG1-CTD and PBD participate in memory maintenance by inhibiting cofilin activity. Our research also indicated that the increases in CaMKⅡ, and cofilin phosphorylation resulting from social interaction occur prior to the increase in NLG1-CTD (Figs. S2f, g and 5j, k), suggesting that earlier changes, such as the activation of NMDA receptors and Rac1, may also be involved in the phosphorylation of these proteins.^48,49^ Considering that both α-secretase and γ-secretase have many other substrates,^19,50,51^, and vHPC NRXN1 also play mild role in social memory stabilization, we cannot completely rule out the possibility that the proteolysis and proteolysis products of these proteins are also involved in maintaining social memory.

Next, we found that inhibiting the activity of α-secretase in the vHPC blocked the formation of short-term social memory in mice. In contrast, inhibiting the activity of γ-secretase did not affect the formation of social memory but disrupted its maintenance. These results suggest that the formation of short-term social memory may depend on the proteolysis of some key synaptic proteins in the vHPC, thereby disrupting existing synaptic organization or trans-synaptic connections. The shedding effect of α-secretase may facilitate the reconstruction of synaptic composition or structures, which are essential for establishing new synaptic connections. This finding is consistent with previous reports indicating that α-secretase is involved in synaptic plasticity and memory.^52^ Additionally, our results revealed that social interaction promotes cofilin phosphorylation (deactivation) and spine growth and maturation, which are regulated by NLG1-CTD produced by γ-secretase. Meanwhile, inhibiting cofilin phosphorylation impaired the maintenance of social memory and the restorative effect of the Tat-PBD peptide. These findings suggest an intriguing mechanism by which social stimuli induce both intracellular and extracellular remodeling of ventral hippocampal synaptic structure, which play distinct roles in the formation and maintenance of social memory, respectively. The product (NLG1-CTD) of sequential cleavage, initiated by extracellular shedding, further inhibits intracellular cytoskeletal remodeling (by cofilin), thereby regulating synaptic plasticity^53,54^ and the maintenance of social memory.

Finally, we found that the social memory maintenance loss in sequential social tasks may be due to the insufficient production of NLG1-CTD. When the time interval between two social objects was short (2 min), the memory of the second social object could be formed but not well maintained, while the memory of the first social object was both formed and maintained normally. However, when the interval was extended to 10 min, the test mice were able to maintain their memory of the second stranger mouse. These results suggest that there is a distinct temporal effect in the maintenance of social memory for different objects. Specifically, when an individual sequentially interacts with two different social objects and the time interval between the two is short enough, the memory of the first social object will be stronger than or suppress the memory maintenance of the second one, resembling the primacy effect observed in serial position effects.^42–44^ Through WB and spine morphology analyses, we found that when test mice interacted with different social partners within a brief time window, neither NLG1-CTD levels nor spine strength in the vHPC showed any further increase. We further demonstrated that supplementation with the PBD peptide overcomes the plateau in dendritic spine maturation and rescues the impairment in memory maintenance associated with exposure to a second novel mouse. These results suggest that sequential social interactions or learning events occurring within a short interval may occupy or deplete molecular or spine structural resources dependent on NLG1 proteolysis, thereby impairing the encoding of subsequent social information. This leads to suppressed NLG1-CTD generation and compromises the persistence of social memory. However, the precise molecular and cellular mechanisms underlying this process remain to be elucidated.

In summary, this study uncovered a novel mechanism by which social interaction promotes the proteolysis of NLG1 and the generation of NLG1-CTD in the ventral hippocampus, which is essential for maintaining social memory. Our discovery elucidates a physiological mechanism that links memory formation, memory maintenance, and LTP maintenance. Additionally, this study may offer an explanation for the primacy effect in sequential social tasks and provides new insights for the treatment of cognitive-related brain disorders.

## Materials and methods

### Mice

NLG1 knockout (KO) mice were sourced from the Jackson Laboratory (Stock: 008136). Genotyping of the NLG1 KO mice was performed using standard PCR techniques, as previously described.^23^ The floxed *Nlg1* (NLG1^*f/f*^) and *Nrxn1* (NRXN1^*f/f*^) mice were generated by Gempharmatech Co., Ltd (Jiangsu, China), with the strategy of LoxP sites flanking the third exon of the *Nlg1* gene and the 18th exon of *Nrxn1* gene through CRISPR-Cas9 technology, respectively. Amigo2-Cre mice were sourced from the Jackson Laboratory (Strain #: 030215). All mice utilized in this study possessed a pure C57BL/6 J genetic background, effectively eliminating potential effects of genetic variability on the phenotype. All experimental mice were male except for those in Fig. S3 a–d and aged between 2 and 4 months. They were housed in the animal facility and cared for in accordance with protocols approved by the Animal Care Committee of Southeast University, Nanjing, China (experiment number: 20211101004). Mice were grouped 2–5 per cage and maintained on a 12-h light/dark cycle with unrestricted access to food and water.

### Viruses, antibodies, and reagents

The viruses, including rAAV2/9-CaMKⅡα-CRE-mCherry-WPRE-pA (Titer: 3.09 × 10^12^ VG/ml), rAAV2/9-EF1α-DIO-hM4D(Gi)-EGFP-WPREs (Titer: 5.08 × 10^12^ VG/ml), rAAV2/9-EF1α-DIO-NLG1-ΔS -WPRE-hGH-pA (Titer: 5.46 × 10^12^ VG/ml), rAAV2/5-EF1α-DIO-NLG1-ΔPBD-WPRE-pA (Titer: 5.79 × 10^12^ VG/ml), rAAV2/5-CMV-DIO-(EGFP-U6)-shRNA(NLG1)-WPRE-hGH-pA (Titer: 6.56 × 10^12^ VG/ml), rAAV2/5-EF1α-DIO-EGFP-NLG1-CTD-WPREs (Titer: 2.00 × 10^12^ VG/ml), and rAAV2/5-EF1α-DIO-NLG1-Full-WPRE-pA (Titer: 5.75 × 10^12^ VG/ml), were purchased from BrainVTA, Wuhan, China.

rAAV9-NCSP-YFP-2E5 (Titer: 5.55 × 10^12^ VG/ml) and rAAV5-EF1α-DIO-EGFP-NLG1-CTDΔ4 (Titer: 2.63 × 10^12^ VG/ml) were purchased from Brain Case, Shenzhen, China. All viruses were aliquoted and have been stored at −80 °C prior to use.

The primary antibodies include anti-NLG1 (1:1000; Cat# 129013, Synaptic System), anti-Puromycin (1:2000; Cat#MABE343, Millipore), anti-cofilin (1:2000; Cat# 5175S, Cell Signaling Technology), anti-phospho-cofilin (1:2000; Cat# 3313, Cell Signaling Technology), anti-ADAM10 (1:2000; Cat#25900-1-AP, Proteintech), anti-ADAM17 (1:2000; Cat#29948-1-AP, Proteintech), anti-GluA1 (1:2000; Cat# MAB2263, Millipore), anti-phospho-GluA1 (Ser 831) (1:2000; Cat#75574S, Cell Signaling Technology), anti-NLG3 (1:2000; Cat# HPA003183, Sigma-Aldrich),anti-HA (1:2000; Cat# 51064-2-AP, Proteintech), anti-CaMKⅡ (1:2000;Cat# ab134041, abcam), anti-phospho-CaMKⅡ (Thr286) (1:2000; Cat# 12716, Cell Signaling Technology), anti-pan NRXN1 (1:2000; Cat# ABN161-I, Millpore), anti-MAP2 (1:250, Cat# 17490-1-AP, Proteintech) and anti-GAPDH (1:4000; Cat# 60004-1-Ig, Proteintech). The secondary antibodies include: goat anti-rabbit (1:2000; Cat# SA00001-2, Proteintech), goat anti-mouse (1:2000; Cat# SA00001-1, Proteintech), Cy3–conjugated Goat Anti-Rabbit IgG(H + L) (1:1000, Cat# SA00009-2, Proteintech), and Alexa Fluor 488 Goat Anti–rabbit IgG (1:200; Cat# A-11008, Thermo Fisher).

The reagents include MRK-560 (Cat# HY-14174, MCE), KN-62 (Cat# A8180, APEXbio), GI254023X (Cat# S8660, Selleck), Anisomycin (Cat# B6674, APExBIO), NBQX (Cat# N171, Sigma-Aldrich), Picrotoxin (PTX, Cat# R284556, Sigma-Aldrich), AP5 (Cat# 0106, Tocris), 1.25% Tribromoethanol (Cat# M2920, EasyCheck), DMEM (Cat# D6429, Sigma-Aldrich), FBS (Cat# 12003 C, Sigma-Aldrich), Lipofectamine 3000 (Cat# L3000150, Thermo Fisher), Paraformaldehyde (Cat# A500684, Sangon Biotech), Clozapine N-oxide (Cat# HY-17366, MedChemExpress), Saline (Cat# CZ0034, Leagene), PBS (Cat# 311-010-CL, Wisent), Streptavidin-conjugated FITC (Cat# SA1001, Thermo Fisher), Mounting medium (Cat# P0131-25ml, Beyotime), Mineral oil (Cat# BS927, Biosharp) and TAT-PBD/REV/S3 peptides (Genscript).

### Social memory test

The social memory tests used in this study were established and modified based on the classic three-chamber social memory experiment. Before the start of the behavioral experiments, all mice were acclimated in the test chamber for at least 60 min. After completing the behavioral test for one mouse, the experimental equipment was wiped with 75% alcohol before proceeding with the next mouse. Testing resumed once the equipment had been allowed to air out fully to minimize the impact of scent on experimental results. The dimensions of the three-chamber apparatus are 60 × 40 × 22 cm, constructed from white opaque acrylic sheets. It is divided into left, middle, and right chambers, with each measuring 20 × 40 × 22 cm. Each partition between the chambers features a small door that connects the adjacent chambers. The stranger mice were matched in age with the subject mice, were selected, and housed separately.

The social memory test comprises several stages, each lasting 10 min. In the first stage, the experimental mouse was placed in the apparatus to freely explore for 10 min to acclimate. In the second (social interaction) stage, an unfamiliar mouse, Stranger 1 (S1), was confined on one side of the apparatus by a cylindrical metal grid, allowing the subject mouse to freely explored for another 10 min. For social memory formation test, the third stage involves closing the partition doors, confining the subject mouse to the middle chamber for another 10 min of exploration. And for social memory maintenance test, we sellected 60 min time point as the transitional window between short- and long-term memory, and the subject mouse was directly moved to its homecage for 60 min, or 40 min after the social formation test. In the final stage, another unfamiliar mouse, Stranger for test (SFT or SFT’), was similarly confined on the opposite side, after which the doors are reopened for a further 10 min of exploration. A camera captures the free exploration of the subject mouse during all the stages. In the social interaction stages in sequential social experiment, the stranger mouse, S1 or S2, was confined on the middle chamber by the same cylindrical metal grid. At the social memory test stages, they were confined on the one side-chamber randomly, while SFT or SFT’ mice were placed in the other side-chamber.

The social memory index is calculated as follows: (Sniffing time with SFT or SFT’- Sniffing time with S1 or S2) / (Sniffing time with SFT or SFT’ + Sniffing time with S1 or S2).

### Novel object recognition

Experimental mice were allowed to adjust to the quiet environment before testing. During the test, each mice was placed in an open-field box (40 × 40 × 40 cm) and allowed to move freely for 10 min. Next, two identical objects (red cube) were placed in the box, and the mouse explored them for another 10 min. After a 1-h interval, one of the objects was replaced with a novel object (blue cylinder) differing in both color and shape. The mice was then placed back into the open field, and the time spent sniffing the novel and familiar objects was recorded during a 5-min test session. After each test, the open field and the objects were cleaned with 75% ethanol. A camera captures the free exploration of the subject mouse during all the stages.

The formula used to determine the discrimination index was (Exploring time with novel − Exploring time with familiar)/(Exploring time with novel + Exploring time with familiar).

### Western blot analysis

The mouse brain was promptly dissected, and the ventral hippocampus was homogenized in a cold lysis buffer containing 20 mM Tris (pH 7.5), 150 mM NaCl, 1 mM EDTA, 1 mM EGTA, 0.5% NP-40, 2.5 mM pyrophosphate, 1 mM β-glycerophosphate, 1 mM Na_3_VO_4_, 20 mM NaF, and 1% protease and phosphatase inhibitors (Thermo Fisher, Product No. A32961). For cytoplasmic protein preparing, NP-40 was excluded from the lysis buffer. Lysis occurred on ice for 40 min with sufficient physical grinding. After centrifugation at 12,000 rpm for 10 min at 4 °C, the supernatant was collected and mixed with a 5× SDS buffer (10% SDS, 0.5% bromophenol blue, 50% glycerol, 250 mM Tris-HCl, 5% β-mercaptoethanol, pH 7.4) and heated in a 98 °C metal bath for 10 min. The sample was subsequently centrifuged and stored either on ice or at −20 °C. The prepared protein samples underwent electrophoresis on SDS-PAGE gels and were transferred to PVDF membranes, which were blocked in 5% nonfat dry milk in TBST (9% NaCl, 20 mM Tris, 1% Tween-20, pH 7.6). Primary antibodies were diluted in TBST and incubated at 4 °C for 12 h. After washing, the membranes were incubated with secondary antibodies for 1 h, followed by further washing and chemiluminescent detection (Thermo Fisher, Cat# 34577). For Coomassie Blue staining, upon completion of protein gel electrophoresis, the gel was immersed in Coomassie Brilliant Blue solution (1.2 g/L Coomassie Brilliant Blue R-250, 50% methanol, 10% acetic acid, 40% ddH_2_O) for 2 h and then washed with elution buffer (10% glacial acetic acid, 30% formylic acid, 60% ddH_2_O) until the background was clean. Protein bands were analyzed using AlphaEaseFC software, and the levels of target proteins were normalized to the immunoreactivity of GAPDH for quantitative analysis.

For all protein collection experiments following social or novel object stimulation, animals were carefully handled and habituated at the testing environment—a clean, empty cage containing an empty pencil holder (for social stimulation) for at least 10 min prior to stimulation. After this habituation period, a stranger mouse or novel object was gently introduced for 10 min before removal. The test mice remained in the same cage for varying durations before sacrifice and sample collection.

### Mass spectrometry analysis

The mass spectrometry experiments were conducted at the Nanjing Jiangbei New Area Biopharmaceutical Public Service Platform (Nanjing, China). In brief, proteins were extracted from the PAGE gel and subsequently digested into peptide fragments using trypsin. The resulting peptides were then purified and separated using the EASY-nLC 1200 high-performance liquid chromatography (HPLC) system (Thermo Fisher). Following this, the peptides were eluted, ionized, and analyzed by tandem mass spectrometry with Q Exactive HF-X instrument (Thermo Fisher). The resulting mass spectrometry data were processed and analyzed using Proteome Discoverer (v2.4).

### Stereotaxic surgery

The detailed methods for stereotactic surgery have been previously described. Mice were anesthetized by intraperitoneal injection of 1.25% tribromoethanol. A glass electrode filled with mineral oil and attached to a 10 µL Shanghai Gaoge microsyringe was used injected to the virus. A microinjection pump and its controller (RWD, Shenzhen, Cat# D01476-002) were employed to control the injection speed at 0.05 µL/min. The needle was gradually lowered to the target site and maintained in position for 10 min following the injection. Following surgery, mice were rested on a heating pad until full recovery from anesthesia was observed. Using the Bregma as the reference point, the coordinates for bilateral virus injection targeted the ventral CA1 region (AP: −3.16 mm, ML: ±3.33 mm, DV: −4.19 mm), the dorsal CA1 region (AP: −2.18 mm, ML: ±2.00 mm, DV: −1.5 mm) and the dorsal CA2 region (AP: −1.60 mm, ML: ±1.60 mm, DV: −1.7 mm). Mice with virus or peptide injection leakage due to technical issues were excluded after post hoc analysis.

### Intracerebral cannula drug injections

Mice, aged 2–3 months, were used for bilateral hippocampal cannula implantation (RWD, Shenzhen, Cat# 62003, O.D. 0.48 mm, I.D. 0.34 mm) for drug injection experiments. For these experiments, mice were initially anesthetized with 4% isoflurane, then positioned in a stereotaxic apparatus and maintained under 1% isoflurane anesthesia. Each mouse was administered a bilateral injection of 1 µL of drug at a rate of 0.5 µL/min, followed by a 2-min hold post-injection. Mice were then placed on a heating pad until they fully recovered from anesthesia. According to the experimental protocol, behavioral tests were conducted 30 or 60 min after recovery. Specific drugs and their concentrations included: MRK-560 (0.1 mM, 1 µL), KN-62 (0.02 mM, 1 µL), GI254023X (0.1 mM, 1 μL), Anisomycin (0.1 mM, 1 µL), CNO (0.5 mg/kg), and TAT-peptides (0.1 mM, 1 µL).

### Slice electrophysiology

Electrophysiological experiments were performed on the ventral hippocampal slices on mice aged 2-5 months. The mouse brains were quickly removed, and horizontal ventral hippocampal slices (300 μm) were prepared (Leica, VT1000S) in ice-cold artificial cerebrospinal fluid (ACSF) containing (in mM): 120.0 NaCl, 3.0 KCl, 1.2 MgSO_4_, 1.0 NaH_2_PO_4_, 26.0 NaHCO_3_, 2.0 CaCl_2_, 11.0 D-glucose saturated with 95% O_2_/5% CO_2_. After recovery for at least 2 hours at 28°C in saturated ACSF, the slices were transferred to a submersion chamber perfused with the same ACSF. All the recordings were performed at room temperature. The hippocampus and neurons were visualized and recorded using an infrared differential interference contrast microscope (Olympus ×51). Evoked synaptic transmission was induced by electrical stimulation at ventral hippocampal Schaffer collateral pathway and recorded with glass pipettes (3-7 MΩ) filled with ACSF. For input-output curves recording, the stimulus intensity was increased gradually from 0 to 0.5 mA, 1 mA, 2 mA, and 3 mA, and fEPSPs were recorded. To evoke LTP, the fEPSP was recorded at 0.067 Hz, and high-frequency stimulation (HFS, 4 pulses at 100 Hz, each lasting 1 s with 9 s interval) was delivered after at least 20 min stable fEPSP responses. For the whole-cell recordings, the glass pipettes were filled with the intracellular solution containing (in mM) 130.0 CsMeSO_4_, 5.0 NaCl, 1 MgCl_2_, 0.05 EGTA, 10.0 HEPES, 3.0 Mg-ATP, 0.3 Na_3_GTP, and 5.0 QX-314 (pH 7.35) (280–300 mOsm). For mEPSC recording, vCA1 neurons were clamped at −70 mV under voltage clamp model, and incubated in the ACSF containing 100 μM picrotoxin. For mIPSC recording, vCA1 neurons were clamped at 0 mV under voltage clamp model, and incubated in the ACSF containing 20 μM NBQX and 50 μM AP5. During whole-cell recordings, cell series resistance was monitored throughout experiments by applying a (−3 mV) step at the end of each sweep, and the experiment was excluded from analysis if the resistance changed by more than 20%. In all electrophysiological experiments, *n* represents the number of slices or neurons. Normally, 1–2 slices per animal and 3–4 neurons per animal were used. All data acquisition and analysis were done using pCLAMP 10.2 (Molecular Devices) or Mini Analysis software (Synaptosoft).

### HEK cell culture and transfection

HEK293T cells were cultured in DMEM supplemented with 10% FBS. Cells were grown to 80% confluence in 12-well plates before being transiently transfected with Lipofectamine 3000 according to the manufacturer’s protocols. Cells were then maintained for 48 h at 37 °C before being harvested for biochemical analysis or immunostaining.

### Immunohistochemistry

In brief, male mice were anesthetized using 1.25% tribromoethanol and fixed on a foam board with limbs secured using needles. After exposing the heart, a needle was inserted into the left ventricle for perfusion with 0.01 M PBS. Once the liver turned pale, the perfusion was switched to 4% PFA. The brain was then removed, fixed in 4% PFA for 16 hours, and transferred to 30% sucrose for dehydration at 4 °C for 48 hours. The brain was subsequently embedded in Tissue-Tek O.C.T. Compound (Sakura), flash-frozen in liquid nitrogen, and stored at −80 °C. Coronal cryosections of 35 µm thickness were prepared and stored at −20 °C using a Leica CM1950 cryostat.

For biotinylated peptide detection, brain sections were mounted onto glass slides coated with poly-D-lysine and permeabilized with 0.01 M PBS containing 0.1% Triton X-100 (PBST) for 2 h. Sections were blocked with 10% FBS in PBST for 1 h and incubated with Streptavidin-conjugated FITC at 37 °C for 2 h and then washed six times with PBST (5 min each). Finally, sections were mounted using an anti-fade mounting medium containing DAPI. To determine the subcellular localization of biotinylated peptides in neurons, brain sections were mounted onto glass slides coated with poly-D-lysine and permeabilized with 0.01 M PBS containing 0.1% Triton X-100 (PBST) for 2 h. Sections were then blocked with 10% FBS in PBST for 1 h, followed by incubation with streptavidin-conjugated FITC at 37 °C for 2 h. After six washes with PBST (5 min each), the sections were incubated with anti-MAP2 antibody at 4 °C for 16 h and then washed six times with PBST (5 min each). Subsequently, the sections were incubated with Cy3–conjugated Goat Anti-Rabbit IgG(H + L) for 2 h and washed six times with PBST (5 min each). Finally, sections were mounted using an anti-fade mounting medium containing DAPI. For the non-permeable experiments, brain sections were mounted onto glass slides coated with poly-D-lysine and permeabilized with 0.01 M PBS (without Triton X-100) for 2 h. After blocking with 10% FBS in PBS for 1 h, the sections were incubated with streptavidin-conjugated FITC at 37 °C for 2 h and washed six times with PBS (5 min each). The sections were then incubated with 0.01 M PBST (containing 0.1% Triton X-100) for further MAP2 staining, as described above.

For dendritic spine morphology analysis, the secondary dendrites of YFP sparsely labeled vCA1 neurons were imaged using a Zeiss LSM900 confocal microscope. A 20× air objective was utilized for wide-field scanning, while a 63× oil objective with 0.5 μm z-stack intervals was employed for high-resolution spine analysis. Fiji software was used for 3D reconstruction, with dendritic spines defined as protrusions greater than 0.6 μm in length. Spines with a head/neck ratio >1 were classified as mature. For each neuron, at least three 25 μm dendritic segments were analyzed.

For surface HA staining in HEK293T cells, the 4% PFA fixed cells were blocked with 5% FBS in 0.01 M PBS for 1 h and incubated with the anti-HA antibody overnight at 4 °C followed by 3 times of wash with PBS. Cells were then incubated with appropriate secondary antibody for 1 h at room temperature. After washing with PBS for 5 times, coverslips were mounted with mounting medium containing DAPI. Images were captured using a Zeiss LSM 900 confocal microscope, and data were analyzed using Fiji software.

### Statistical analysis

Statistical analyses and data visualizations were conducted using Prism 9 (GraphPad). All averaged data are expressed as the mean ± standard error of the mean (SEM). Statistical evaluations included two-tailed unpaired and paired Student’s t-tests, as well as one-way or two-way ANOVA. Results were deemed statistically significant at p < 0.05 (*p < 0.05, **p < 0.01, ***p < 0.001), with “ns” denoting non-significant outcomes (p > 0.05).

## Supplementary information

Supplementary Figures

## Data Availability

All data supporting the findings of this study are available within the main text and Supplementary Information. Source data are provided with this paper as a Source Data file. All data are available from the corresponding author upon request. Source data are provided with this paper.
