# Supplementary material for: Social memory maintenance relies on social interaction-induced proteolytic products of neuroligin 1
Source: Signal Transduct Target Ther. 2025 Nov 24;10:387. doi: 10.1038/s41392-025-02467-6 (PMC12641031; doi:10.1038/s41392-025-02467-6)
Supplement: Supplementary file 1 — Supplementary Figures [file 41392_2025_2467_MOESM1_ESM.docx]

Supplementary Materials for

**Social Memory Maintenance Relies on Social Interaction-Induced Proteolytic Products of Neuroligin 1**

An Liu^1,2,#^, Xingcan Li^1,2,#^, Mei Zhuang^1,2^, Qiaoyun Ren^1,2^, Jinglei Zhang^1,2^, Dandan Lv^1,2^, Miao Wu^1,2^, Xingjie Bian^1,2^, Chengyan Zhu^1,2^, Xiuqi Yang^1,2^, Moyi Li^1,2^, Yanan Wang^1,2^, Zhengping Jia^1,3,4,*^ and Wei Xie^1,2,5*^

Correspondence to: Wei Xie at [wei.xie@seu.edu.cn](mailto:wei.xie@seu.edu.cn)

Zhengping Jia at [zhengping.jia@sickkids.ca](mailto:zhengping.jia@sickkids.ca)

**This PDF file includes:**

Figures. S1 to S9

**Supplementary Figures**


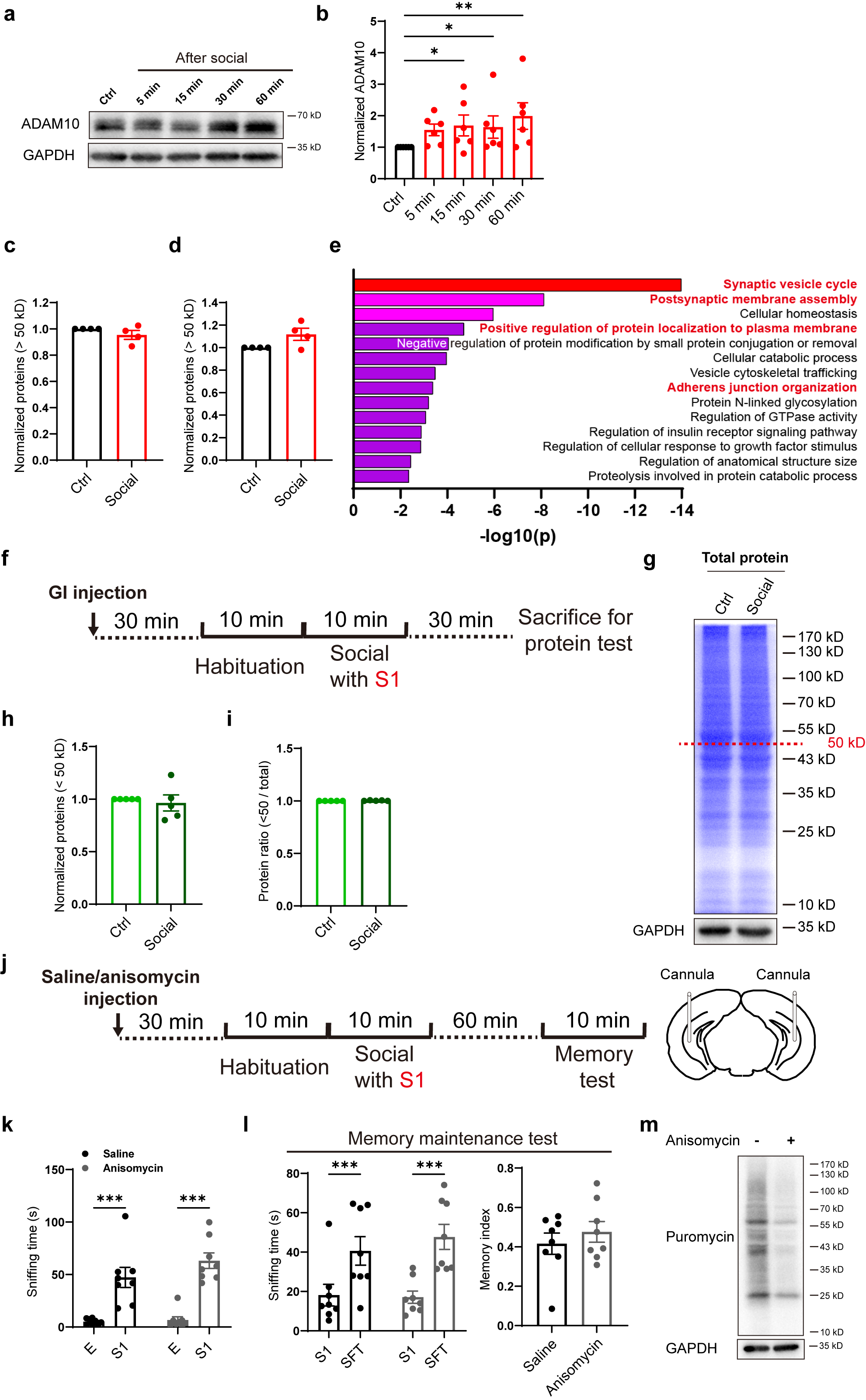


**Figure S1. Social interaction activates ADAM10 and promotes proteolysis (Related to Figure 1).**

**a-b**. Sample images and summary graphs showing increased ADAM10 (n = 6) in mice vHPC tissues after social interaction;

**c-d**. Summary graphs showing normal >50 kDa proteins in total (**c**) and cytosolic (**d**) vHPC tissues of social group (n = 4 mice);

**e**. GO enrichment analysis of the increased proteins in **Figure 1i**;

**f**. Illustration of drug in situ injection and collecting mice vHPC tissues protein test;

**g**. Sample image of Coomassie blue staining of total vHPC proteins after GI pre-treatment;

**h-i**. Summary data showing comparable total vHPC <50 kDa proteins (**h**) and protein ratio (**i**) in GI pre-treated Ctrl and social groups (n = 5 mice);

**j**. Illustration of Anisomycin injection and social memory maintenance detection;

**k**. Sniffing time detection showing both the saline (n = 8) and Anisomycin (n = 8) injected mice preferred S1 over empty cage (E);

**l**. Sniffing time detection (left) and memory maintenance index (right) showing both the saline (n = 8) and Anisomycin (n = 8) injected mice preferred SFT’ over S1;

**m**. Pruomycin staining showing reduced new protein synthesis in Anisomycin pre-treated mouse.

Data represent mean ± SEM; two-tailed t-test for right panels of **c**, **d**, **h**, **i** and right panel of **l**; one-way ANOVA with Fisher's LSD post hoc comparisons for **b**; two-way ANOVA with Fisher's LSD post hoc multiple comparisons for **k** and left panel of **l**. *p < 0.05, ***p < 0.001.


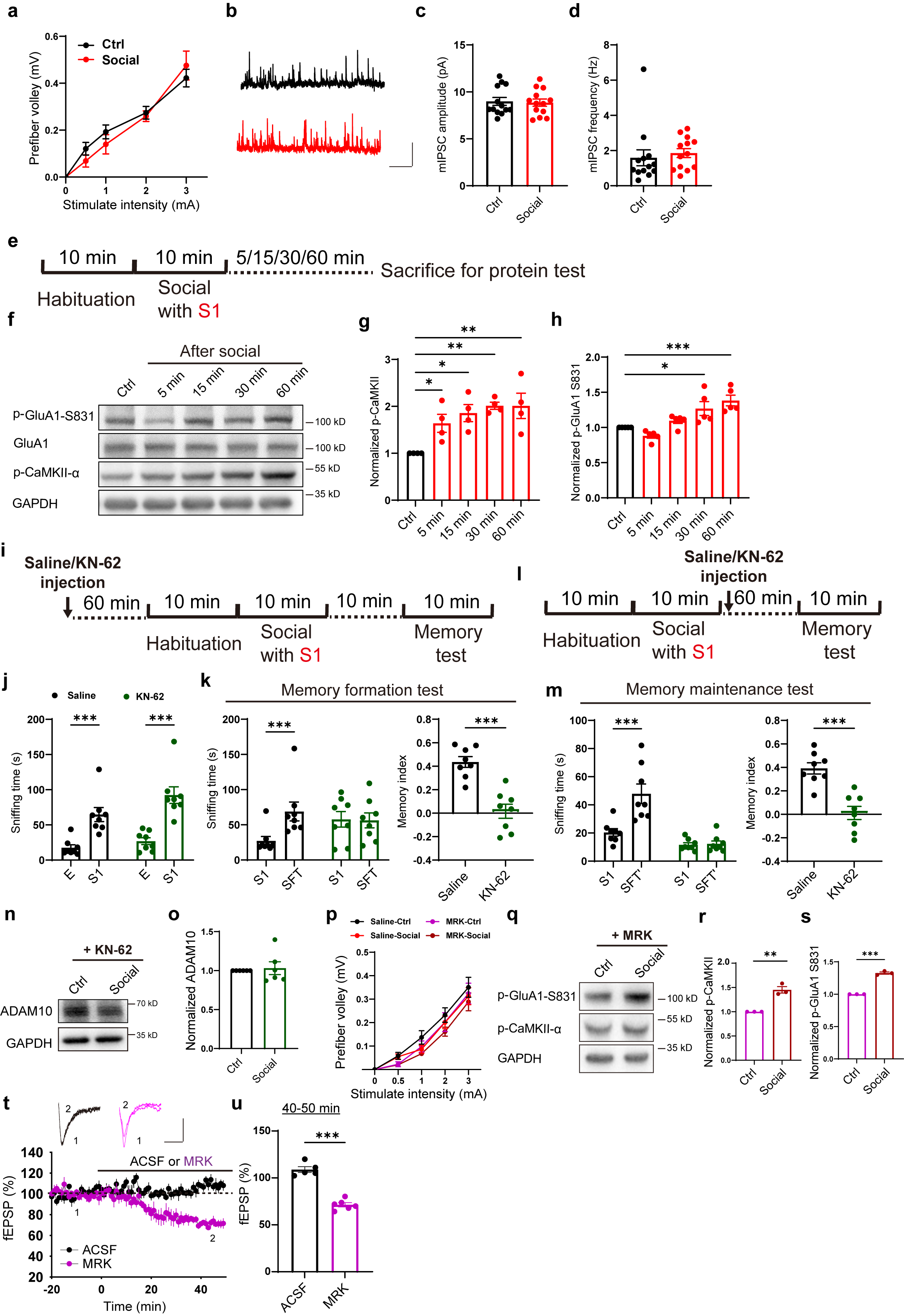


**Figure S2. vHPC CaMKII activity critically regulates social memory dynamics (Related to Figure. 2).**

**a**. Comparable input/output curve of fEPSP prefiber volley in Ctrl and social group of vHPC slices (both n = 14 slices from 5 mice);

**b-d**. Sample traces and summary graphs showing intact mIPSC amplitude (**c**) and frequency (**d**) in the vCA1 neurons of social group (n = 13 cells from 4 mice) compared to that of Ctrl mice (n = 13 cells from 4 mice), scale bar: 10 pA/2 s;

**e**. Illustration of collecting vHPC proteins at different time points after social;

**f-h**. Sample images and summary graphs showing increased CaMKII-α (**g**) and GluA1-S831 (**h**) phosphorylation in vHPC tissues that have undergone social interaction;

**i**. Illustration of drug injection at vHPC and memory test;

**j**. Sniffing time detection showing the saline (n = 8 mice) and KN-62 (n = 8 mice) injected mice preferred S1 over E;

**k**. Sniffing time detection (left) and memory formation index (right) showing saline (n = 8 mice) and KN-62 (n = 8 mice) injected mice preferred SFT over S1;

**l**. Illustration of drug injection at vHPC and memory test;

**m**. Sniffing time detection (left) and memory formation index (right) showing saline (n = 8 mice), but not KN-62 (n = 8 mice) injected mice preferred SFT’ over S1;

**n-o**. Sample images and summary graphs showing intact vHPC ADAM10 (n = 6) in KN-62 pre-injected Ctrl and Social groups of mice at 1h post-social interaction;

**p**. Comparable input/output curve of fEPSP prefiber volley in Saline and MRK pre-treated Ctrl and social groups of vHPC slices (n = 14 slices from 5 mice for each group);

**q-s**. Sample images and summary graphs showing increased CaMKII-α (n = 3 mice, **r**) and GluA1-S831 site (n = 3 mice, **s**) phosphorylation in MRK pre-treated vHPC tissues 1h after social interaction;

**t-u**. Summary graphs showing decreased fEPSP in MRK treated vHPC slices (n = 6 slices from 3 mice) compared to that in Ctrl slices (n = 5 slices from 3 mice), scale bar: 0.5 mV/25 ms.

Data represent mean ± SEM; two-tailed t-test for **c**, **d**, **o**, **r**, **s**, **u** and right panels of **k** and **m**; one-way ANOVA with Fisher's LSD post hoc comparisons for **g**, **h** and left panels of **k** and **m**; repeated two-way ANOVA with Fisher's LSD post hoc multiple comparisons for **a** and **p**. *p < 0.05, **p < 0.01, ***p < 0.001.


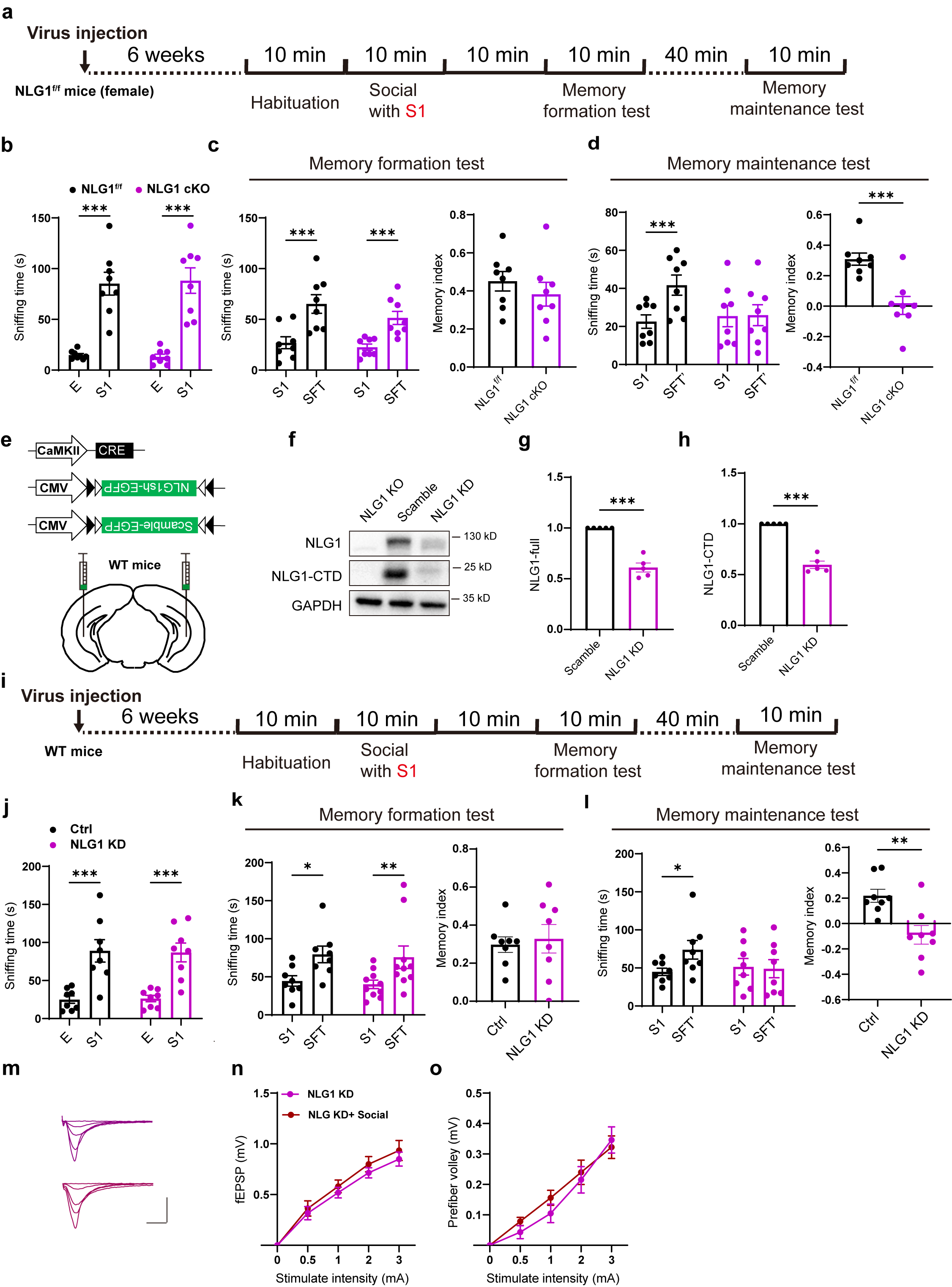


**Figure S3. NLG1 is necessary for social memory maintenance both in male and female mice (Related to Figure 3).**

**a**. Illustration of virus injection at vHPC and social memory formation and maintenance detection in NLG1*^f/f^* female mice;

**b**. Sniffing time detection showing both the female NLG1*^f/f^* (n = 8) and NLG1 cKO (n = 8) mice preferred S1 over E;

**c**. Sniffing time detection (left) and memory formation index (right) showing both the female NLG1*^f/f^* (n = 8) and female NLG1 cKO (n = 8) mice preferred SFT over S1;

**d**. Sniffing time detection (left) and memory maintenance index (right) showing the female NLG1*^f/f^* (n = 8), but not NLG1 cKO (n = 8) mice preferred SFT' over S1;

**e**. Illustration of injecting NLG1sh-EGFP and Scramble-EGFP cDNA containing viruses in WT vHPC excitatory neurons;

**f-h**. Sample images and summary graphs showing decreased full-length NLG1 and NLG1-CTD in NLG1 KD (n = 5) mice;

**i**. Illustration of viruses injection and social memory formation and maintenance detection in male WT mice;

**j**. Sniffing time detection showing both the male Ctrl (n = 8) and NLG1 KD (n = 8) mice preferred S1 over E;

**k**. Sniffing time detection (left) and memory formation index (right) showing both the male Ctrl (n = 8) and NLG1 KD (n = 8) mice preferred SFT over S1;

**l**. Sniffing time detection (left) and memory maintenance index (right) showing the male Ctrl (n = 8), but not NLG1 KD (n = 8) mice preferred SFT' over S1;

**m-o**. Sample traces and summary input/output curves of fEPSPs (**n**) and prefiber volley (**o**) showing intact synaptic transmission in social group (n = 12 slices from 4 mice) compared to the slices from Ctrl NLG1 KD mice (n = 12 slices from 4 mice), scale bar: 0.5 mV/10 ms.

Data represent mean ± SEM; two-tailed t-test for **c**, **d**, right panels of **g** and **h**; two-way ANOVA with Fisher's LSD post hoc comparisons for **f**, left panels of **g** and **h**; repeated two-way ANOVA with Fisher's LSD post hoc multiple comparisons for **j** and **k**. *p < 0.05, **p < 0.01, ***p < 0.001.


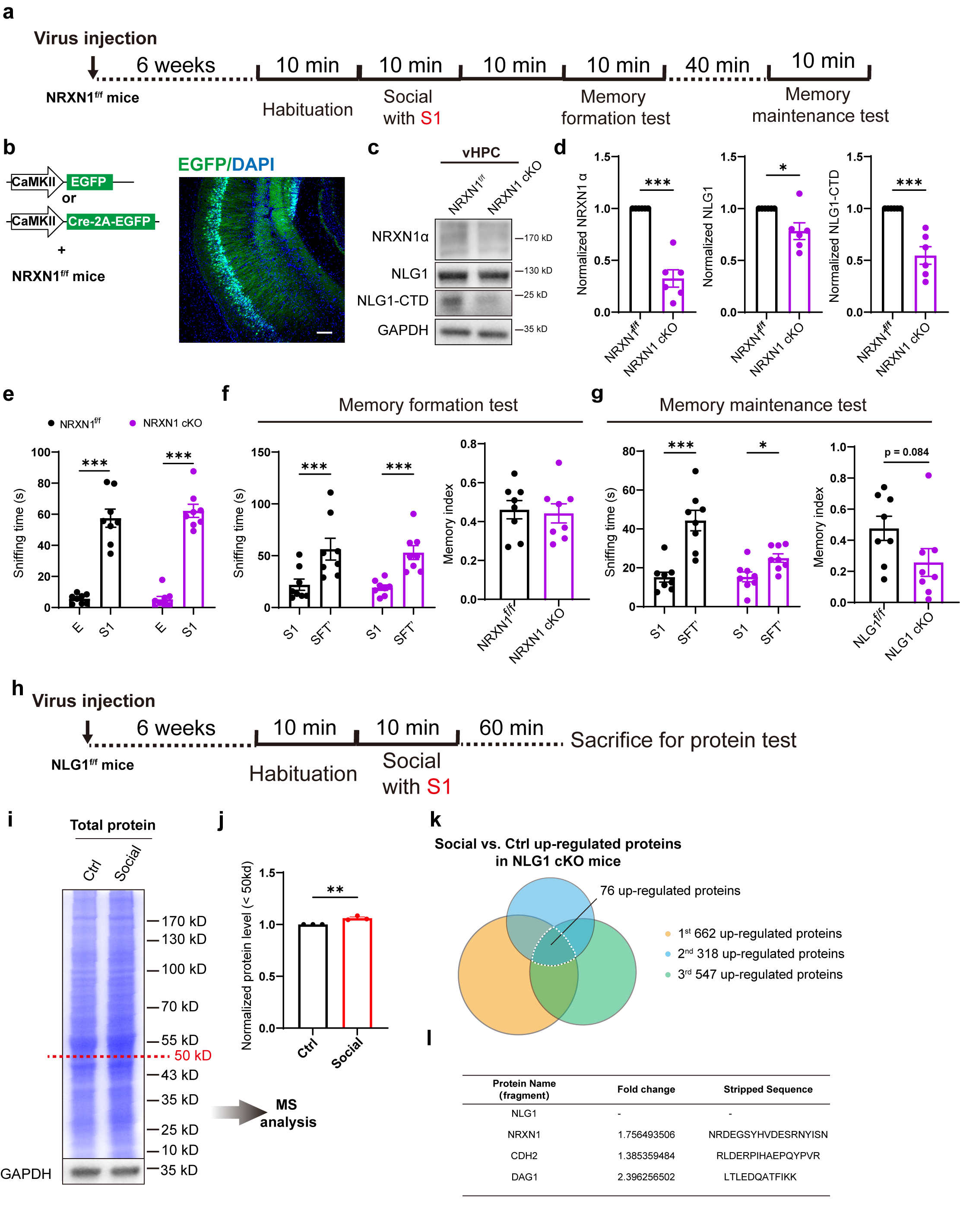


**Figure S4. Conditional knockout of NRXN1 in the vHPCs mildly impairs social memory maintenance (Related to Figure 3).**

**a**. Illustration of virus injection at vHPC and social memory formation and maintenance detection in NRXN1*^f/f^* male mice;

**b**. Illustration and sample image showing injecting and expressing AAV viruses in vHPC of NRXN1*^f/f^* mice, scale bar: 100 μm;

**c-d**. Sample images and summary graphs showing decreased NRXN1α, NLG1 and NLG1-CTD in NRXN1 cKO mice (n = 6);

**e**. Sniffing time detection showing both the NRXN1*^f/f^* (n = 8) and NRXN1 cKO (n = 8) mice preferred S1 over E;

**f**. Sniffing time detection (left) and memory formation index (right) showing both the NRXN1*^f/f^* (n = 8) and NRXN1 cKO (n = 8) mice preferred SFT over S1;

**g**. Sniffing time detection (left) and memory maintenance index (right) showing both the NRXN1*^f/f^* (n = 8) and NRXN1 cKO (n = 8) mice preferred SFT' over S1;

**h**. Illustration of virus injection at vHPC and protein test in NLG1*^f/f^* male mice;

**i**. Sample image of Coomassie blue staining of total vHPC proteins from NLG1 cKO mice;

**j**. Summary data showing significantly increased <50 kDa proteins in total vHPC tissues of social group (n = 3 mice);

**k**. Summary graph of the 3 times of MS analyses showing 76 proteins (fragments) were increased in the social group;

**l**. List of 3 increased proteins (fragments) in (**k**).

Data represent mean ± SEM; two-tailed t-test for **d**, **j** and right panels of **f** and **g**; two-way ANOVA with Fisher's LSD post hoc comparisons for **e** and left panels of **f** and **g**. *p < 0.05, ***p < 0.001.


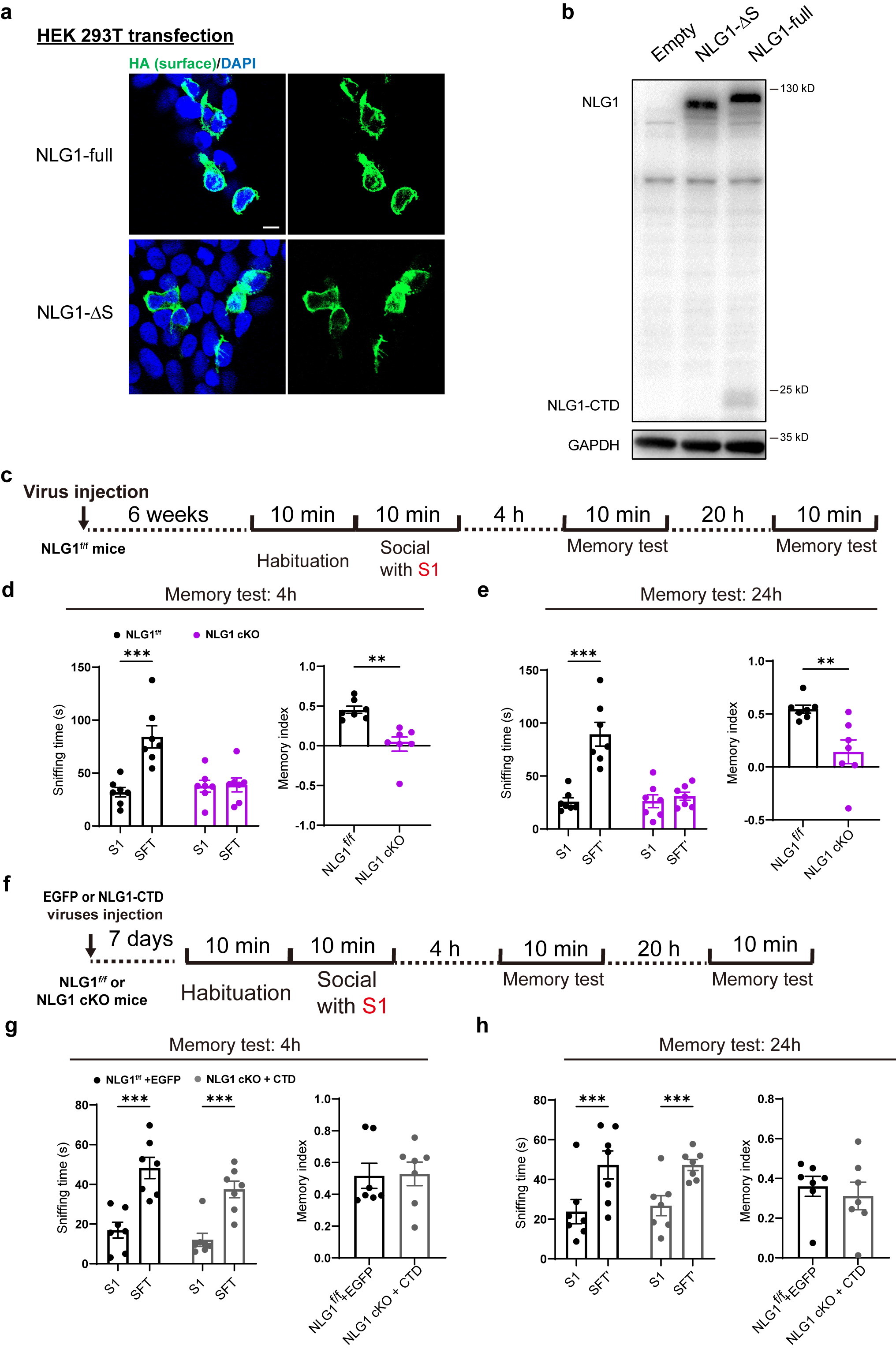


**Figure S5. Supplementation of NLG1-CTD rescues the impairment in social memory maintenance at both 4 and 24 hours in NLG1 cKO mice (Related to Figure 4).**

1. Sample images of surface HA staining in transfected HEK-293T cells showing both NLG1-full and NLG1-δS proteins can be transported to the cell membrane, scale bar: 10 μm;

**b**. Sample images of Western blot showing the lack of NLG1-CTD in NLG1-δS plasmid transfected HEK-293T cells;

**c**. Illustration of virus injection and social memory test 4h and 24h post-interaction in NLG1*^f/f^* mice;

**d**. Sniffing time detection (left) and memory maintenance index (right) showing the NLG1*^f/f^* (n = 8), but not NLG1 cKO (n = 8) mice preferred SFT over S1 4h post-interaction;

**e**. Sniffing time detection (left) and memory maintenance index (right) showing the NLG1*^f/f^* (n = 8), but not NLG1 cKO (n = 8) mice preferred SFT’ over S1 24h post-interaction;

**f**. Illustration of virus injection and social memory test 4h and 24h post-interaction in NLG1*^f/f^* or NLG1 cKO mice;

**g**. Sniffing time detection (left) and memory maintenance index (right) showing both the NLG1*^f/f^* +EGFP (n = 8) and NLG1 cKO +CTD (n = 8) mice preferred SFT over S1 4h post-interaction;

**h**. Sniffing time detection (left) and memory maintenance index (right) showing both the NLG1*^f/f^* +EGFP (n = 8) and NLG1 cKO +CTD (n = 8) mice preferred SFT’ over S1 24h post-interaction.

Data represent mean ± SEM; two-tailed t-test for right panels of **d**, **e**, **g** and **h**; two-way ANOVA with Fisher's LSD post hoc comparisons for left panels of **d**, **e**, **g** and **h**. **p < 0.01, ***p < 0.001.


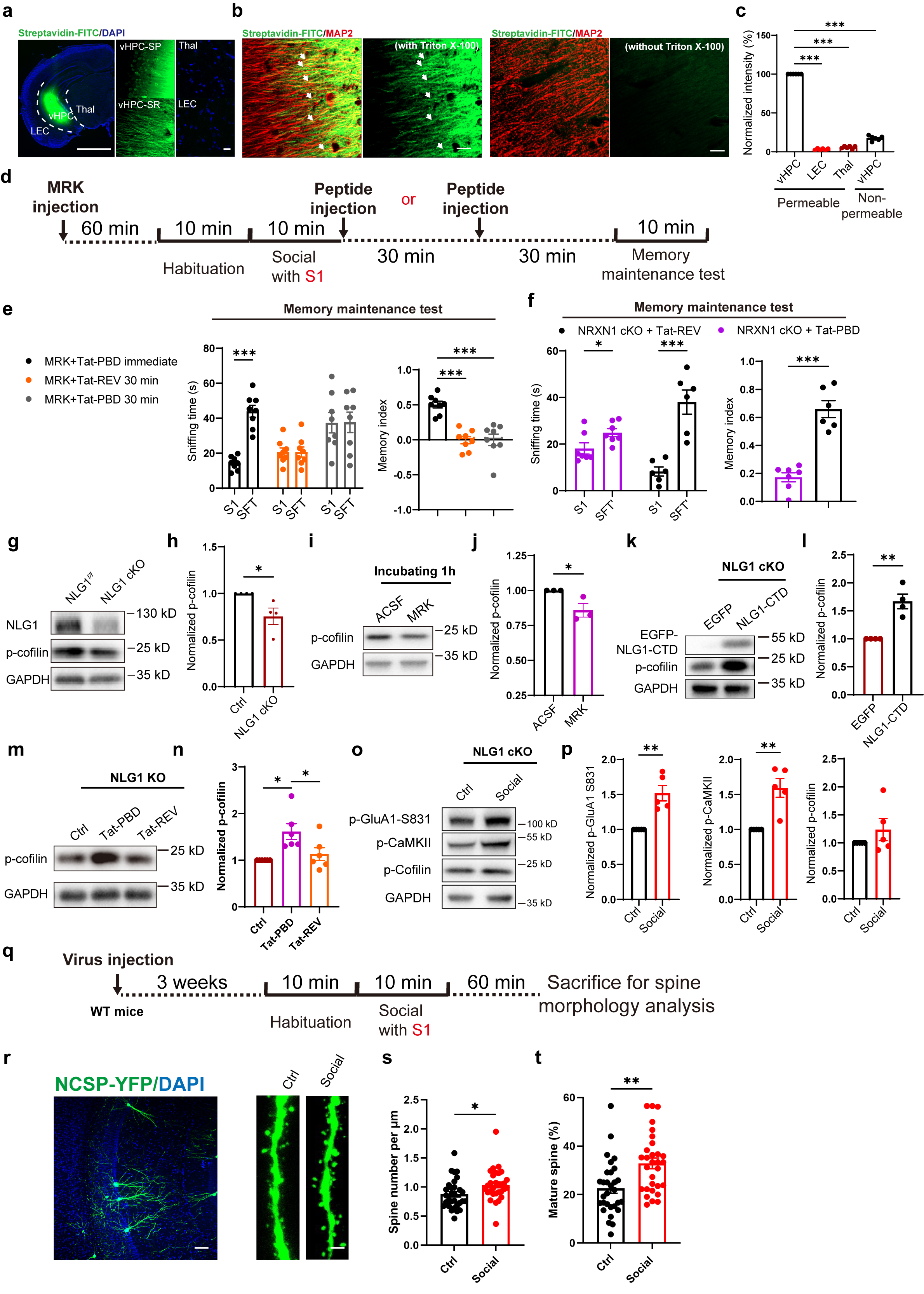


**Figure S6. Tat-PBD peptide promotes social memory maintenance and cofilin phosphorylation (Related to Figure 5).**

1. Sample images show the detection of FITC (Tat-PBD peptide) signals in the vHPC, LEC, and Thal regions at 1 hour after injection of 500 nM Tat-PBD peptide, stratum pyramidal (SP), right scale bar: 500 μm, left scale bar: 20 μm;
2. Sample images show the detection of peptide and MAP2 signals in the permeable and non-permeable vHPC SR regions,scale bar: 20 μm;
3. Summary graph show the normalized FITC intensities in different groups in (**a**) and (**b**);
4. Illustration of drug and peptide injection and social memory test 1h post-interaction in WT mice;
5. Sniffing time detection (left) and memory maintenance index (right) showing the MRK + Tat-PBD-immediately group (n = 8), but not Tat-REV (n = 8) or Tat-PBD (n=8) 30 min-after social interaition groups of mice preferred SFT over S1;
6. Sniffing time detection (left) and memory maintenance index (right) showing the Tat-PBD (n = 6), but not Tat-REV (n = 6) injected NRXN1 cKO mice preferred SFT over S1;

**g-h**. Sample images and summary graph showing decreased p-cofilin in NLG1 cKO (n = 4) mice;

**i-j**. Sample images and summary graph showing decreased p-cofilin in MRK treated vHPC slices (n = 3 mice);

**k-l**. Sample images and summary graph showing increased p-cofilin in NLG1-CTD expressed NLG1 cKO (n = 4) mice;

**m-n**. Sample images and summary graph showing increased p-cofilin in Tat-PBD, but not Tat-REV peptide treated NLG1 KO (n = 6) slices;

**o-p**. Sample images and summary graphs showing increased p-GluA1 S831 and p-CaMKII, but not p-cofilin level in social stimulated NLG1 cKO mice (n = 5);

**q**. Illustration of virus injection and spine morphology analysis in WT mice;

**r**. Sample image of YFP expressed neurons and dendrites in vHPC, left scale bar: 100 μm, right scale bar: 2 μm;

**s-t**. Sample images and summary graphs showing elevated spine density and mature spine ratio in Social group (n = 30 dendrites from 15 neurons of 5 mice) compared to the Ctrl group (n = 30 dendrites from 15 neurons of 5 mice) of vCA1 neurons.

Data represent mean ± SEM; two-tailed t-test for **g**, **j**, **l**, **n**, **p**, **s**, **t** and right panel of **f**; one-way ANOVA with Fisher's LSD post hoc comparisons for **n** and right panel of **e**; repeated two-way ANOVA with Fisher's LSD post hoc multiple comparisons for left panels of **e** and **f**. *p < 0.05, **p < 0.01, ***p < 0.01.


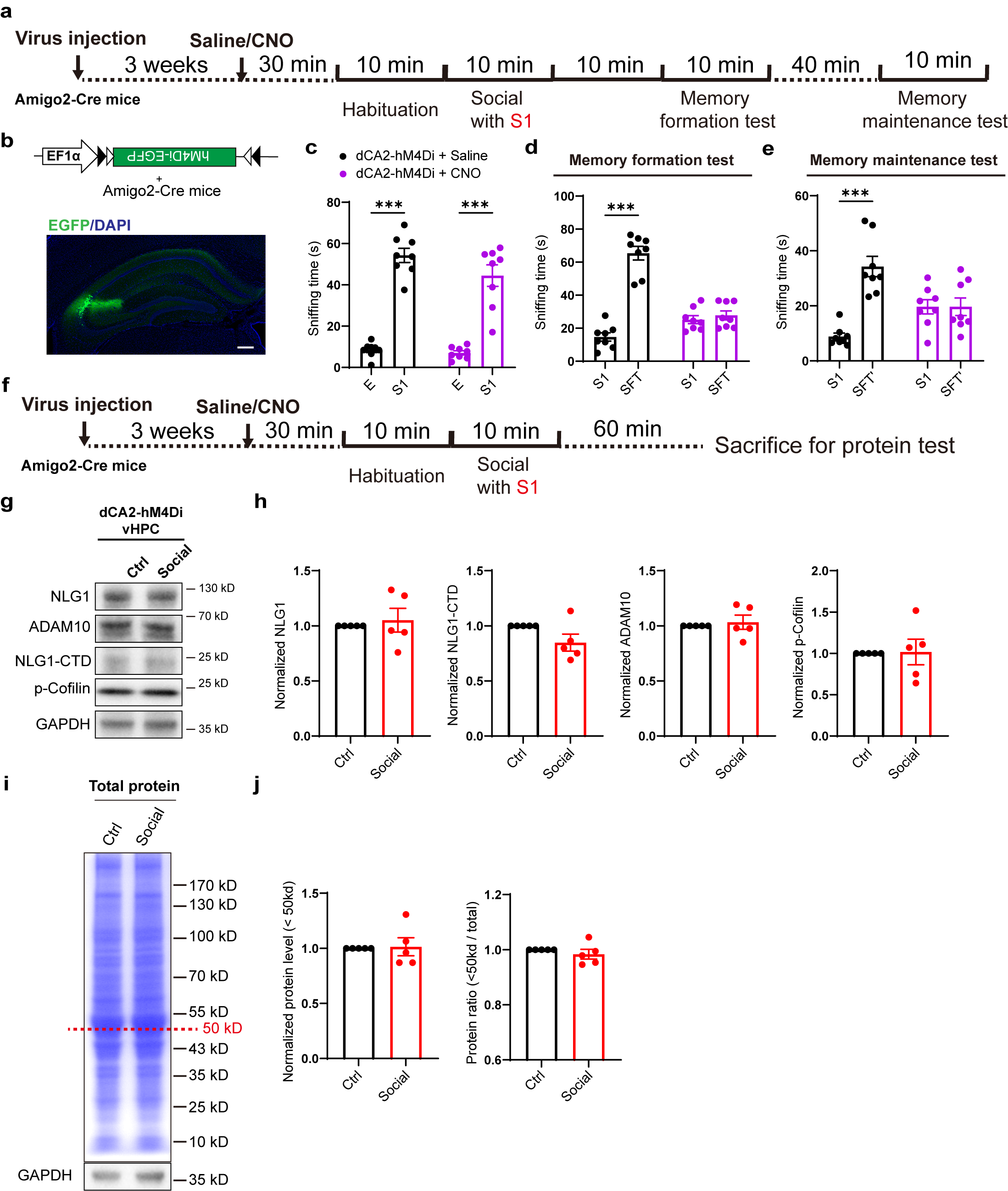


**Figure S7. Inhibition of dCA2 pyramidal neurons prevents social induced ADAM10 activation and proteolysis (Related to Figure 5).**

**a**. Illustration of virus and CNO injection and social memory detection in Amigo2-Cre male mice;

**b**. Illustration and sample image showing expressing hM4Di-EGFP in dCA2 region, scale bar: 100 μm;

**c**. Sniffing time detection showing both the dCA2-hM4Di + Saline (n = 8) and dCA2-hM4Di + CNO (n = 8) mice preferred S1 over E;

**d**. Sniffing time detection (left) showing the dCA2-hM4Di + Saline (n = 8), but not dCA2-hM4Di + CNO (n = 8) mice preferred SFT over S1;

**e**. Sniffing time detection (left) showing the NLG1*^f/f^* (n = 8), but not NLG1 cKO (n = 8) mice preferred SFT' over S1;

**f**. Illustration of virus and CNO injection and protein test in Amigo2-Cre male mice;

**g-h**. Sample images and summary graphs showing intact NLG1, NLG1-CTD, ADAM10 and p-cofilin levels in vHPC tissues from social stimulated dCA2-hM4Di + CNO mice (n = 5);

**i**. Sample image of Coomassie blue staining of total vHPC proteins from control and social stimulated dCA2-hM4Di + CNO mice;

**j**. Summary data showing significantly intact <50 kDa proteins (left) and protein ratio (right) in total vHPC tissues of social group (n = 5 mice).

Data represent mean ± SEM; two-tailed t-test for **h** and **j**; two-way ANOVA with Fisher's LSD post hoc comparisons for **c**, **d** and **e**. ***p < 0.001.


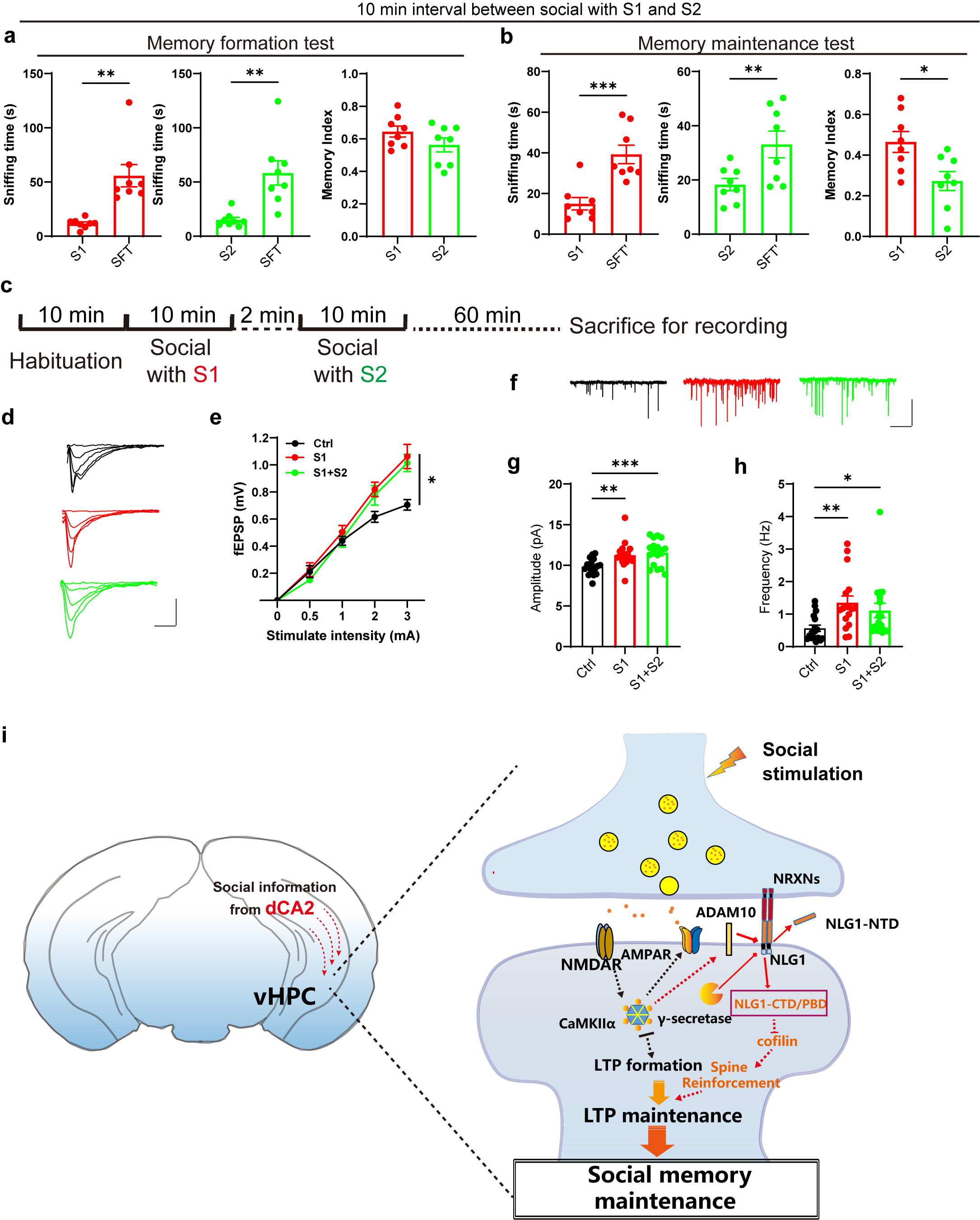


**Figure S8. Exposure to a second social object after a short inverval doesn’t further enhance synaptic transmission in the vHPC** **(Related to Figure 6).**

**a**. Sniffing time detection and memory formation index (right) showing the subject mice (n=8) preferred SFT over S1 (red) and S2 (green) in the 10 min-interval continuous social task;

**b**. Sniffing time detection and memory maintenance index (right) showing the subject mice (n=8) preferred SFT' over S1 (red) and S2 (green) in the 10 min-interval continuous social task, and the memory index for S1 is slightly higher than that for S2 (right panel);

**c**. Illustration of recording on vHPC slices from mice that have undergone 2 min-interval social task;

**d-e**. Sample traces and summary input/out curves showing a similar degree of elevation in the S1-only (n = 15 slices from 5 mice) and 2 min-interval S1+S2 (n = 15 slices from 5 mice) groups, compared with that in the Ctrl group (n = 15 slices from 5 mice), scale bar: 0.5 mV/10 ms;

**f-h**. Sample traces and summary graphs of mEPSC showing increased mEPSC amplitude (**g**) and frequency (**h**) in the vCA1 pyramidal neurons from S1-only (n = 17 cells from 5 mice) and 2 min-interval S1+S2 (n = 17 cells from 5 mice) mice, compared to thouse in Ctrl group (n = 17 cells from 5 mice), scale bar: 10 pA/2 s;

**i**. Working model. Social information from dCA2 region activates α-secretase in the vHPC, which sequentially cleaves NLG1 to generate soluble NLG1-CTD with the involvement of γ-secretase. The NLG1-CTD then inhibits cofilin activity and promotes spine reinforcement, thereby contributing to LTP and social memory maintenance. The red arrows indicate signaling pathways demonstrated in this study, while the black arrows represent those reported in previous publications, solid lines denote direct regulations, and dashed lines represent indirect mechanisms (created with Adobe Illustrator).

Data represent mean ± SEM; paired two-tailed t-test for the first two panels of **a** and **b**; one-way ANOVA with Fisher's LSD post hoc comparisons for **g** and **h**; repeated two-way ANOVA with Fisher's LSD post hoc multiple comparisons for **e**. *p < 0.05, **p < 0.01.


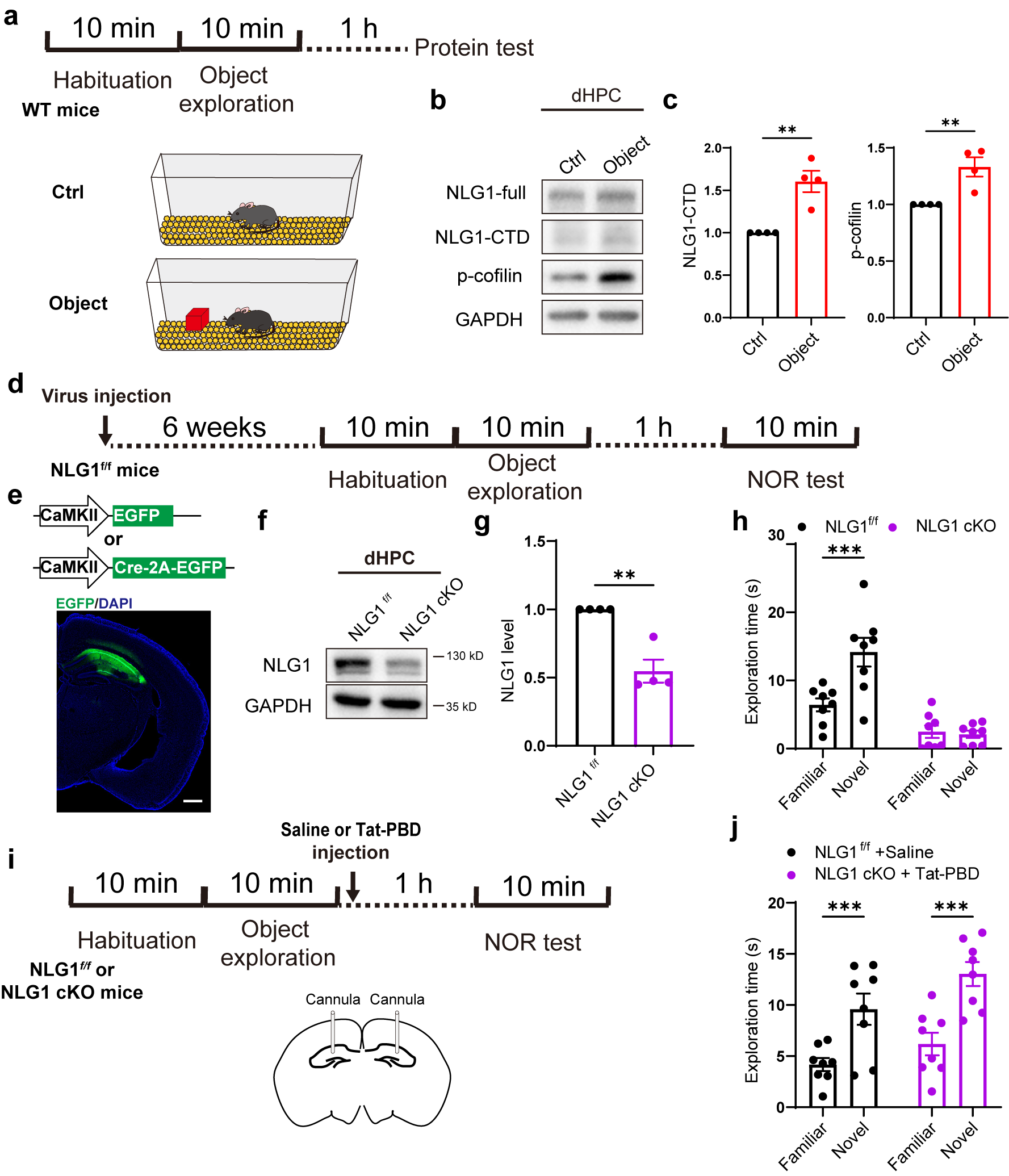


**Figure S9. Tat-PBD peptide rescues NOR maintenance defict in dHPC NLG1 cKO mice.**

**a**. Illustration of novel object exploration and protein analysis in WT mice;

**b-c**. Sample images and summary graphs showing elevated NLG1-CTD and p-cofilin levels in dHPC of object-exploration groups of mice (n = 4);

**d**. Illustration of virus injection and novel object recognition test in NLG1*^f/f^* mice;

**e**. Illustration and sample image showing injecting and expressing AAV viruses in vHPC of NLG1*^f/f^* mice, scale bar: 200 μm;

**f-g**. Sample images and summary graph showing decreased NLG1 in NLG1 cKO mice (n = 4);

**h**. Exploration time detection showing the NLG1*^f/f^* (n = 8), but not NLG1 cKO (n = 8) mice preferred novel over familiar object;

**i**. Illustration of peptide injection at dHPC and novel object recognition test in NLG1*^f/f^* and NLG1 cKO mice;

**j**. Exploration time detection showing both the NLG1*^f/f^* + saline (n = 8) and NLG1 cKO +Tat-PBD (n = 8) mice preferred novel over familiar object.

Data represent mean ± SEM; two-tailed t-test for **c** and **g**, right panels of **f** and **g**; two-way ANOVA with Fisher's LSD post hoc comparisons for **h** and **j**. **p < 0.01, ***p < 0.001.
